# Methicillin-resistant *Staphylococcus* aureus nasal swabs: trends in use and association with outcomes

**DOI:** 10.1017/ash.2025.10093

**Published:** 2025-08-07

**Authors:** Hayley B. Gershengorn, Hannah Wunsch, Bhavarth Shukla

**Affiliations:** 1 Division of Pulmonary, Critical Care and Sleep Medicine, University of Miami Miller School of Medicine, Miami, FL, USA; 2 Division of Critical Care Medicine, Albert Einstein College of Medicine, Bronx, NY, USA; 3 Department of Anesthesiology, Weill Cornell Medical College, New York, NY, USA; 4 Sunnybrook Research Institute, Toronto, ON, Canada; 5 Department of Anesthesiology and Pain Medicine, University of Toronto, Toronto, ON, Canada; 6 Division of Infectious Disease, Department of Medicine, University of Miami Miller School of Medicine, Miami, FL, USA

## Abstract

**Objective:**

To investigate patterns of early methicillin-resistant *Staphylococcus aureus* (MRSA) nasal swab use in US hospitals and the association with de-escalation of MRSA-specific antibiotics.

**Design:**

Retrospective cohort study.

**Setting:**

PINC-A1 Healthcare Database (2008–2021).

**Participants:**

Adults with sepsis present on admission who received invasive mechanical ventilation by hospital day 1.

**Methods:**

We assessed interhospital variation and time trends in early polymerase chain reaction-based MRSA nasal swab use using bivariable regression. Next, we used competing risks multivariable regression to assess the association of early (started by hospital day 2) anti-MRSA antibiotic duration with care in a high (≥90%) versus low (<10%) swab use hospital.

**Results:**

We included 699,474 patients across 788 hospitals to evaluate trends in early swab use; 151,205 (21.6%) received a swab. Use of swabs varied across hospitals (median use: 6.0% [interquartile range: 0–37.6%; full range: 0%–98.0%]; median odds ratio [95% CI]: 84.7 [63.3–115.6]) and overall use increased over time (3.5% in 2008 quarter 1 increasing to 29.5% in 2021 quarter 4; regression coefficient [95% CI]: 0.14% [0.12%–0.15%]). Considering 41,599 patients (9,796 [23.6%] in 33 hospitals where ≥90% received swabs and 31,763 [76.4%] in 67 hospitals with <10% use), anti-MRSA antibiotic durations were shorter in hospitals where ≥90% (vs < 10%) received a swab (adjusted sub-hazard ratio for discontinuation of antibiotics [95% CI]: 1.17 [1.04–1.31], *P* = .007).

**Conclusions:**

Use of early polymerase chain reaction-based MRSA nasal swabs varied across US hospitals and increased over time. Receiving care in a hospital with higher swab use was associated with shorter anti-MRSA antibiotic duration.

## Introduction

*Staphylococcus aureus* is a common pathogen in the intensive care unit.^1,2^ The proportion of *S. aureus* isolates resistant to methicillin has been rising,^3^ resulting in guidelines recommending the use of empiric antibiotics with activity against methicillin-resistant *S. aureus* (MRSA).^4^ Therefore, quickly and accurately ruling out MRSA infection in critically ill patients is of high importance to minimize overuse of these medications.

Evaluating for colonization with MRSA is possible using nasal swabs with rapid (<3 d) detection of the organism.^5^ A meta-analysis of 22 studies found MRSA nasal screening was 70.9% sensitive for the diagnosis of MRSA pneumonia which, assuming a 10% prevalence, led to a negative predictive value of 96.5%.^6^ The negative predictive value of MRSA nasal swabs for other MRSA infections is less well studied, but appears similarly high.^5^ Across >500,000 patients hospitalized in the Veterans Affairs system, MRSA nasal swabs had a negative predictive value of 96.5% for any MRSA infection.^7^

Existing evidence on the impact of MRSA nasal swab testing on anti-MRSA antibiotics durations comes from a handful of small (n = 79–2 910) single-center studies.^8–16^ While most suggest use of MRSA nasal swabs may reduce anti-MRSA antibiotic use, duration, and associated costs, not all confirm these findings. Moreover, no study focused on critically ill patients in whom decisions to de-escalate antibiotics may be considered riskier.

Thus, we sought to investigate the use of polymerase chain reaction (PCR)-based MRSA nasal swab testing and its association with the duration of anti-MRSA antibiotics across a large, US multicenter cohort of critically ill adults. We hypothesized that use would vary across hospitals, but increase over time, and that critically ill septic patients cared for in hospitals that used PCR-based MRSA swabs more commonly would receive anti-MRSA antibiotics for shorter durations.

## Methods

We conducted a retrospective cohort study of critically ill adults (≥18 yr) discharged from US hospitals in the PINC-A1 Healthcare Database from 2008 to 2021.

### Cohorts

To explore the use of PCR-based MRSA nasal swabs across hospitals and over time (2008–2021), we focused on a population with critical illness due to infection likely causing their hospitalization. We included patients with sepsis (using the Angus et al International Classification of Diseases 9^th^ [ICD-9] and 10^th^ [ICD-10] revisions criteria for infection present on admission and organ dysfunction at any time during hospitalization) who received invasive mechanical ventilation (MV; at least one ICD-9/ICD-10 and one charge code for MV^17^) by hospital day 1. Patients were excluded if they received an MRSA nasal swab (determined by charge code) prior to hospital day 0, were transferred in from another hospital, or were discharged from the hospital prior to hospital day 2 (to mitigate against immortal time bias). Patients were then excluded if they were discharged from hospitals with <100 patients remaining in the cohort (to allow better assessment of hospital-level practices).

To evaluate the association of PCR-based MRSA swab use and anti-MRSA antibiotics duration, we further excluded patients discharged prior to 2016 (to include a more current cohort [2016–2021], all defined by ICD-10 codes). We then excluded patients who did not receive an anti-MRSA antibiotic (defined as intravenous vancomycin, oral/intravenous linezolid, or intravenous ceftaroline) by hospital day 2 or were discharged from a hospital in which no cohort patients received an MRSA nasal swab (out of concern for miscoding at those sites). Again, we then excluded patients discharged from hospitals with <100 remaining cohort patients.

### Exposures and outcomes

For our analyses considering usage trends, we considered two co-primary exposures: individual hospital and discharge quarter (three-month period). Our outcome was MRSA nasal swab (by polymerase chain reaction) use by hospital day 2 to represent colonization evaluation early during hospitalization and infection. MRSA swab use was determined by patient-level charges for swabs (charges are provided in the PINC-A1 Healthcare Database chargemaster for all items [eg, medications, room and board, laboratory tests] billed to a patient indexed by hospital day); thus, only swabs where costs were assigned to the patient were captured.

For our analysis evaluating the association of hospital-level PCR-based MRSA nasal swab use with outcomes, our primary exposure was discharge from a hospital in which ≥90% (vs < 10%) of cohort patients received a swab by hospital day 2. Our primary outcome was consecutive days of anti-MRSA antibiotics. We also assessed two secondary outcomes: consecutive days of first MV case and hospital mortality. For the subgroup who did not receive dialysis by hospital day 2, we *post hoc* assessed new use of dialysis (continuous or intermittent) after hospital day 2.

### Statistical analyses

We used standard statistics to describe cohort characteristics. We compared patients receiving MRSA nasal swabs and not, as well as across hospital-level exposure categories, using standardized mean differences (SMDs).

We quantified early MRSA nasal swab use across hospitals and over time by percentages with 95% confidence intervals (CIs). We assessed the difference across hospitals using mixed effects logistic regression (dependent variable = patient-level receipt of an early MRSA nasal swab; no fixed effects; random effect = individual hospital) and the median odds ratio. The presence of a trend in use over time was assessed using univariable linear regression (independent variable = discharge quarter; dependent variable = percentage of cohort during that quarter with an MRSA nasal swab).

To evaluate the association between hospital-level early MRSA nasal swabs and anti-MRSA antibiotic duration among patients started on such medications by hospital day 2, we used competing risks multivariable regression. Our primary outcome was modeled as the discontinuation of all anti-MRSA antibiotics (defined by a single calendar day without them) with a competing risk of in-hospital death. Covariables are listed in eTable 1. Standard errors were clustered by hospital. We used a similar model structure for first MV duration (modeled as discontinuation of MV) and a Cox proportional hazards model for hospital mortality. Finally, we used Kruskal-Wallis testing to compare hospital-level use of anti-MRSA antibiotics across MRSA nasal swab use deciles.

We also performed several sensitivity analyses. First, we reconstructed our primary model for duration of anti-MRSA antibiotics using three sensitivity exposures: deciles (<10%, 10 to <20%, …≥90%; preplanned); restricted cubic splines with 4 knots (preplanned); and linear (*post hoc*). We used Wald testing of all exposure variables (categories and spline terms) to assess the association of the exposure overall with anti-MRSA antibiotic duration and Wald testing of the higher-order spline terms to assess for nonlinearity. Second, we reran our primary model using our primary exposure, but across four preplanned sensitivity cohorts. Given concerns that patients with renal injury could receive a “continuous” course of anti-MRSA antibiotics that was intentionally not administered on consecutive days (and, thus, their initial duration would be misclassified), we first excluded patients who ever received dialysis during their hospitalization and then, additionally, patients with renal organ dysfunction. We next expanded our primary cohort to any patients with early sepsis (irrespective of early MV) and then, those with early MV (irrespective of early sepsis).

*Post hoc*, we explored the association of hospital-level MRSA nasal swab use with a “negative control outcome,”^18^ the duration of piperacillin-tazobactam and/or cefepime antibiotics, reasoning that such antibiotics should not be directly affected by MRSA nasal swab testing.

All analyses were performed using Stata MP 18.5 (College Station, TX) and Microsoft Office 2024 (Redmond, WA). This study was considered exempt from institutional board review by the University of Miami. *P* values of <.05 and SMDs ≥.2 were considered statistically significant.^19^ No adjustments were made for multiple comparisons; thus, all non-primary analyses are considered exploratory.

## Results

### Epidemiology of early PCR-based MRSA nasal swab use

**Figure 1.**
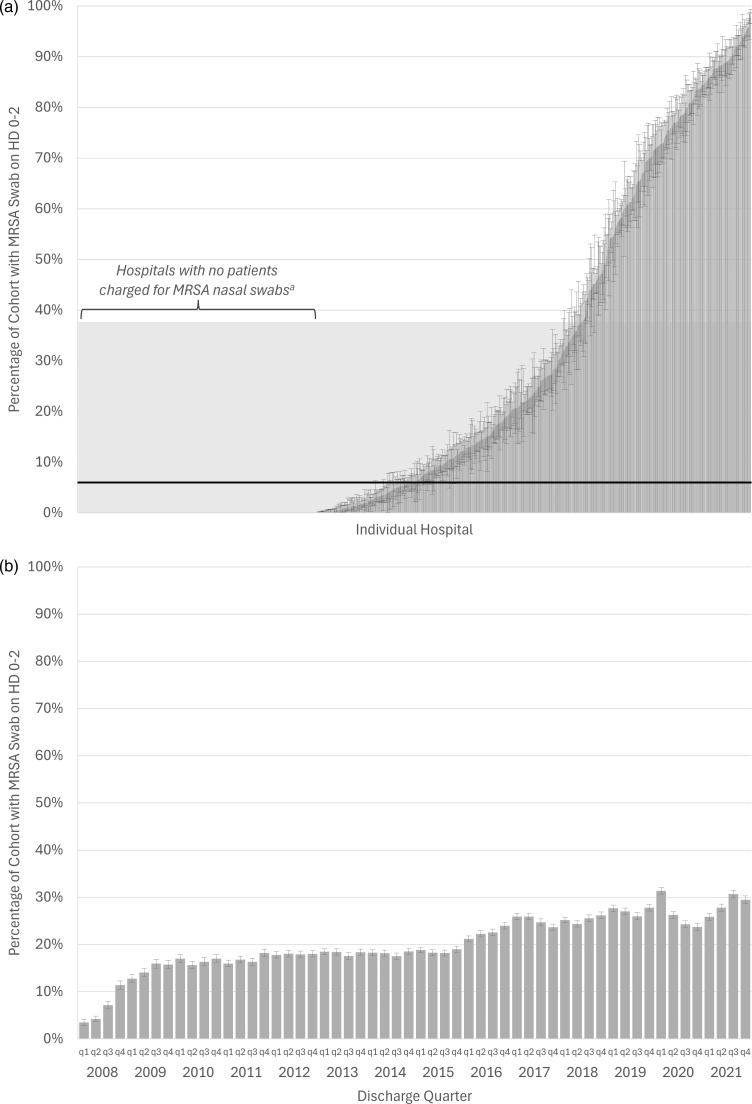
Trends in early MRSA nasal swab use. (A) Across individual hospitals. (B) By discharge quarter HD, hospital day; MRSA, methicillin-resistant *staphylococcus aureus* bars = % with 95% CI; panel A: black line = median, shaded area = p25 – p75; panel A: mixed effects model with median odds ratio = 84.7 (63.3–115.6); panel B: linear regression across quarters with .14% (.12%–.15%) increase per quarter *a* Plausibly no patients received swabs or, possibly, screening was universal and not charged to patients; median use across hospitals excluding these non-use hospitals: 23.8%.

Our primary cohort used to evaluate use of MRSA nasal swabs early during hospitalization among patients with sepsis and MV receipt included 699,474 patients across 788 hospitals (Figure 1). Of these, 151,205 (21.6%) received an MRSA nasal swab by hospital day 2. Patients who received swabs did not differ from those who did not across any measured demographic or illness characteristics (Table 1, Table 2). Patients who received early MRSA nasal swabs (vs not) were more commonly cared for in hospitals in the Northeast (18.7% of those with MRSA swabs vs 13.4% without) or West (22.1% vs 16.8%) of the United States, and less commonly in the Midwest (18.7% vs 21.2%) or South (40.5% vs 48.5%, SMD = .224). Hospital teaching status, hospital size, and urbanicity were similar across groups.

**Table 1.** Characteristics for cohort evaluating trends in early MRSA nasal swab use

	no MRSA swab, N (%)	MRSA swab, N (%)	SMD
# of patients, N (row%)	548,269 (78.4)	151,205 (21.6)	n/a
Age, mean (sd)	63.5 (15.5)	63.6 (15.4)	.007
Female gender^a^	268,055 (48.9)	72,7651 (48.1)	.015
Race			.103
White	375,239 (68.4)	109,6931 (72.5)	
Black	93,823 (17.1)	24,4331 (16.2)	
Other/unknown^b^	79,207 (14.4)	17,0791 (11.3)	
Payor			.045
Private	74,771 (13.6)	19,2661 (12.7)	
Medicare	343,232 (62.6)	95,0331 (62.9)	
Medicaid	90,088 (16.4)	26,7161 (17.7)	
Other	40,178 (7.3)	10,1901 (6.7)	
Elixhauser comorbidity #	5.9 (2.4)	6.0 (2.4)	.037
Major surgery	99,918 (18.2)	25,8881 (17.1)	.029
Resource use			
Vasopressors by HD 2	246,993 (45.0)	68,4101 (45.2)	.004
Dialysis by HD 2	37,974 (6.9)	10,4791 (6.9)	<.001
Acute organ dysfunctions			
Renal	258,631 (47.2)	75,4901 (49.9)	.055
Neurologic	192,576 (35.1)	56,6541 (37.5)	.049
Liver	35,487 (6.5)	10,2261 (6.8)	.012
Hematologic	99,465 (18.1)	28,0571 (18.6)	.011
Cardiovascular	263,956 (48.1)	78,3531 (51.8)	.074
Discharge year			.299
2008	15,356 (2.8)	1,1441 (.8)	
2009	21,570 (3.9)	3,7041 (2.4)	
2010	26,830 (4.9)	5,3151 (3.5)	
2011	35,811 (6.5)	7,2491 (4.8)	
2012	39,087 (7.1)	8,5491 (5.7)	
2013	43,851 (8.0)	9,7771 (6.5)	
2014	46,399 (8.5)	10,2851 (6.8)	
2015	47,714 (8.7)	10,8941 (7.2)	
2016	46,785 (8.5)	13,5481 (9.0)	
2017	49,282 (9.0)	16,5341 (10.9)	
2018	47,273 (8.6)	16,0071 (10.6)	
2019	45,611 (8.3)	17,0071 (11.2)	
2020	46,190 (8.4)	16,7591 (11.1)	
2021	36,510 (6.7)	14,4331 (9.5)	
Teaching hospital	273,165 (49.8)	73,6341 (48.7)	.023
Hospital bed #			.150
500+	190,746 (34.8)	48,9121 (32.3)	
400–499	74,053 (13.5)	18,9691 (12.5)	
300–399	108,516 (19.8)	25,1601 (16.6)	
200–299	98,886 (18.0)	31,4461 (20.8)	
100–199	63,278 (11.5)	23,1461 (15.3)	
0–99	12,790 (2.3)	3,5721 (2.4)	
Hospital region			.224
Midwest	116,484 (21.2)	28,2211 (18.7)	
Northeast	73,658 (13.4)	28,3061 (18.7)	
South	266,009 (48.5)	61,2371 (40.5)	
West	92,118 (16.8)	33,4411 (22.1)	
Urban hospital	497,744 (90.8)	134,6911 (89.1)	.057

HD, hospital day; MRSA, methicillin-resistant *Staphylococcus aureus*; sd, standard deviation; SMD, standardized mean difference.

a the PINC-A1 Healthcare Database uses the term “gender” rather than “sex”; thus, we have used the same throughout.

b includes “Hispanic ethnicity” as ethnicity was not a separate category prior in the data set prior to 2012 (and, thus, patients could be coded as “Hispanic” for race) and this cohort includes patients discharged prior to this time.

**Figure 2.**
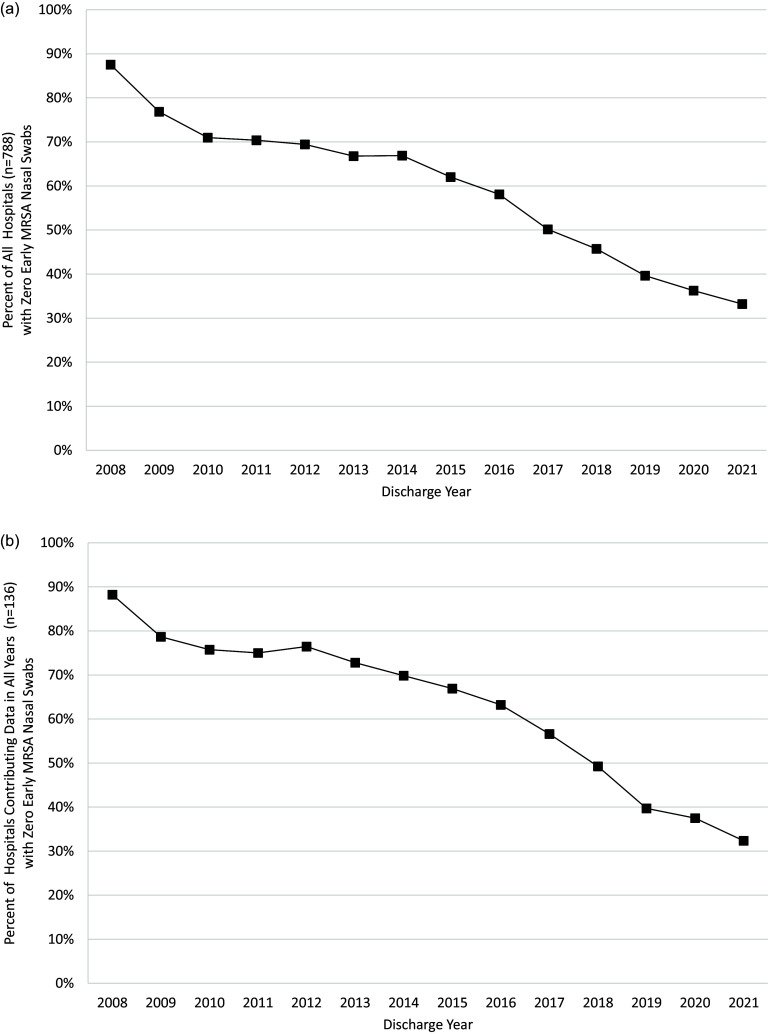
Trends in hospitals reporting zero use of early MRSA nasal swabs over time. (A) All hospitals (n = 788). (B) Only hospitals contributing at least one patient to cohort in all years (n = 136). MRSA, methicillin-resistant *staphylococcus aureus*.

Use of early MRSA nasal swabs varied across hospitals (Figure 1A, median odds ratio [95% CI]: 84.7 [63.3–115.6]). The median hospital used an early MRSA nasal swab on 6.0% of cohort patients (interquartile range [IQR]: 0%–37.6%; full range: 0%–98.0%). Over the 14-year period, 178 (22.6%) hospitals did not use early MRSA nasal swabs on any patients (as defined by patient-level swab charges), yet such non-use hospitals represented a smaller proportion of the cohort over time (Figure 2). Patients in hospitals with no use of early MRSA swabs based on patient charges were similar to patients in hospitals with at least one swab; however, a larger proportion of them came from hospitals in the Midwest (26.8% vs 17.6%) and West (20.6% vs 16.6%) regions of the US (SMD .286; Tables 3 and 4). Overall use of early MRSA nasal swabs increased over time (Figure 1B, regression coefficient [95% CI]: .14% [.12%–.15%]) from 3.5% of cohort patients in quarter 1 of 2008 to 29.5% in quarter 4 of 2021.

### Association of hospital-level early PCR-based MRSA swab use and outcomes

Our primary outcomes cohort consisted of 41,599 patients, 9,796 (23.6%) cared for in 33 hospitals where ≥90% of cohort patients received early MRSA nasal swabs and 31,763 (76.4%) in 67 hospitals where <10% did (Table 2, Table 5). Patient characteristics were largely similar across groups, although patients cared for in hospitals where ≥90% received a swab were more commonly of White race (78.6% vs 69.1%, SMD = .217). Fewer patients in hospitals where ≥90% received a swab were in teaching hospitals (28.0% vs 52.3%, SMD = .512), larger hospitals (500+ beds: 7.0% vs 45.6%, SMD = 1.332), the South US region (31.0% vs 70.3%, SMD = .920), or urban settings (82.5% vs 92.1%, SMD = .291).

**Table 2.** Characteristics for cohort evaluating association of early MRSA nasal swab use with outcomes

	Full Cohort, N (%)	In Hospitals with <10% Early MRSA Nasal Swabs, N (%)	In Hospitals with ≥90% Early MRSA Nasal Swabs, N (%)	SMD^a^
# of patients, N (row%)	41,559 (100.0)	31,763 (76.4)	9,796 (23.6)	
# of hospitals, N (row %)	100 (100.0)	67 (67.0)	33 (33.0)	
Age, mean (sd)	63.4 (15.3)	63.1 (15.3)	64.4 (15.1)	.087
Female gender^b^	19,471 (46.9)	14,874 (46.8)	4,597 (46.9)	.002
Race				.217
White	29,648 (71.3)	21,950 (69.1)	7,6981 (78.6)	
Black	6,768 (16.3)	5,601 (17.6)	1,1671 (11.9)	
Other/unknown^c^	5,143 (12.4)	4,212 (13.3)	9 311 (9.5)	
Hispanic ethnicity	3,788 (9.1)	3,103 (9.8)	685 (7.0)	.100
Payor				.078
Private	5,312 (12.8)	4,072 (12.8)	1,2401 (12.7)	
Medicare	26,616 (64.0)	20,258 (63.8)	6,3581 (64.9)	
Medicaid	6,985 (16.8)	5,276 (16.6)	1,7091 (17.4)	
Other	2,646 (6.4)	2,157 (6.8)	4 891 (5.0)	
Elixhauser comorbidity #	6.3 (2.4)	6.3 (2.4)	6.4 (2.4)	.041
Major surgery	7,950 (19.1)	6,114 (19.2)	1,8361 (18.7)	.013
Resource use				
Vasopressors by HD 2	20,344 (49.0)	15,010 (47.3)	5,3341 (54.5)	.144
Dialysis by HD 2	3,408 (8.2)	2,590 (8.2)	8 181 (8.4)	.007
Acute organ dysfunctions				
Renal	23,477 (56.5)	17,843 (56.2)	5,6341 (57.5)	.027
Neurologic	17,588 (42.3)	13,693 (43.1)	3,8951 (39.8)	.068
Liver	3,503 (8.4)	2,687 (8.5)	8 161 (8.3)	.005
Hematologic	9,169 (22.1)	6,941 (21.9)	2,2281 (22.7)	.021
Cardiovascular	25,390 (61.1)	19,375 (61.0)	6,0151 (61.4)	.008
Discharge year				.282
2016	6,266 (15.1)	4,448 (14.0)	1,8 181 (18.6)	
2017	8,278 (19.9)	6,008 (18.9)	2,2701 (23.2)	
2018	7,924 (19.1)	5,795 (18.2)	2,1291 (21.7)	
2019	7,285 (17.5)	5,666 (17.8)	1,6191 (16.5)	
2020	6,694 (16.1)	5,506 (17.3)	1,1881 (12.1)	
2021	5,112 (12.3)	4,340 (13.7)	7 721 (7.9)	
Teaching hospital	19,364 (46.6)	16,620 (52.3)	2,7441 (28.0)	.512
Hospital bed #				1.332
500+	15,175 (36.5)	14,486 (45.6)	6 891 (7.0)	
400–499	4,014 (9.7)	2,709 (8.5)	1,3051 (13.3)	
300–399	7,210 (17.3)	6,518 (20.5)	6 921 (7.1)	
200–299	11,011 (26.5)	5,155 (16.2)	5,8561 (59.8)	
100–199	3,680 (8.9)	2,571 (8.1)	1,1091 (11.3)	
0–99	469 (1.1)	324 (1.0)	1 451 (1.5)	
Hospital region				.920
Midwest	4,173 (10.0)	3,192 (10.0)	9 811 (10.0)	
Northeast	6,751 (16.2)	3,577 (11.3)	3,1741 (32.4)	
South	25,374 (61.1)	22,334 (70.3)	3,0401 (31.0)	
West	5,261 (12.7)	2,660 (8.4)	2,6011 (26.6)	
Urban hospital	37,333 (89.8)	29,252 (92.1)	8,0811 (82.5)	.291

HD, hospital day; MRSA, methicillin-resistant *Staphylococcus aureus*; sd, standard deviation; SMD, standardized mean difference.

a comparison of <10% versus ≥90% early MRSA nasal swab use hospitals.

b the PINC-A1 Healthcare Database uses the term “gender” rather than “sex”; thus, we have used the same throughout.

c does not include “Hispanic ethnicity” as ethnicity was a separate category from race in the data set beginning in 2012 and this cohort is restricted to patients discharged from 2016 to 2021.

Rates of initiating anti-MRSA antibiotics by hospital day 2 across all patients (n = 66,681) in these 100 hospitals did not differ (median [IQR]: 60.7% [52.5%, 69.1%] for hospitals with ≥90% vs 62.0% [54.2%, 67.9%] with <10% swab use, *P* = .79; Figure 2). Yet, the first duration of anti-MRSA antibiotics for those receiving them was shorter for patients cared for in hospitals where ≥90% of patients received a swab (median [IQR]: 2 [1,3] vs 2 [1,4] days, *P* < .001).

**Figure 3.**
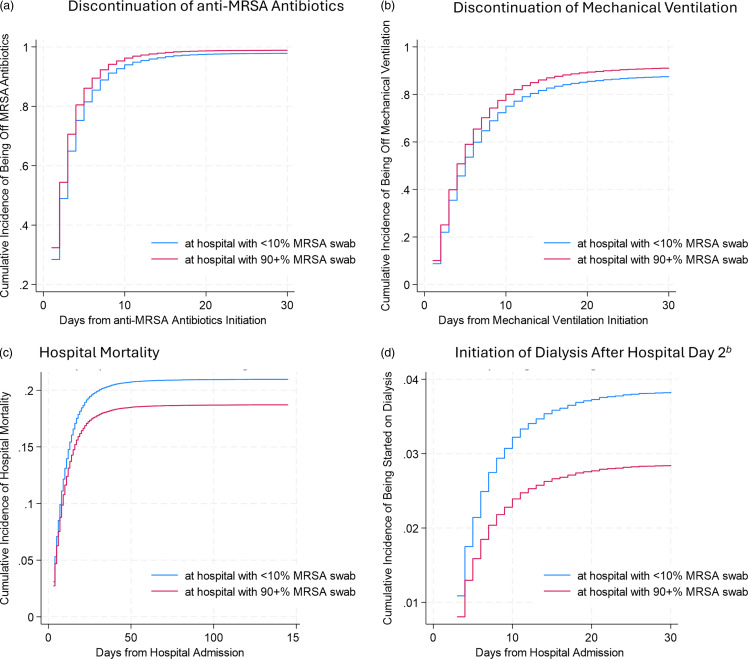
Association of hospital-level early MRSA nasal swab use and outcomes.^*a*^ (A) Discontinuation of anti-MRSA antibiotics. (B) Discontinuation of mechanical ventilation. (C) Hospital mortality. (D) Initiation of dialysis after hospital day 2^*b*^ CI: confidence interval; HR, hazard ratio; MRSA, methicillin-resistant *Staphylococcus aureus*; SHR, sub-hazard ratio; *a* using multivariable competing risks models (except mortality which is time-to-death); Panel A: SHR = 1.17 (95% CI: 1.04, 1.31), *P* = .007; Panel B: SHR = 1.16 (.98, 1.37), *P* = .08; Panel C: HR .88 (.77, 1.01), *P* = .07; Panel D: SHR = .74 (.61, .89), *P* = .001; *b* restricted to patients who did not receive dialysis on or before hospital day 2.

After adjustment for potential confounders and accounting for the competing risk of death, the first duration of anti-MRSA antibiotics remained shorter among patients cared for in hospitals where ≥90% received an early swab (sub-hazard ratio [SHR] for discontinuation of antibiotics [95% CI]: 1.17 [1.04–1.31], *P* = .007; Figure 3A, eTable 6). Similarly positive numeric associations were identified with preplanned secondary outcomes, but these did not reach statistical significance: duration of first MV (SHR for discontinuation [95% CI]: 1.16 [.98–1.37], *P* = .08; Figure 3B) and hospital mortality (hazard ratio for mortality [95% CI]: .87 [.76–1.01], *P* = .06; Figure 3C). The hazard of receiving dialysis after hospital day 2 (among the subgroup without previous receipt) was lower for patients cared for in hospitals where ≥90% received an early MRSA nasal swab (SHR [95% CI]: .74 (.61–.89), *P* = .001; Figure 3D).

Sensitivity analyses considering all deciles of hospital-level early MRSA nasal swab use (eFigure 3A) and restricted cubic splines for percent use demonstrated similarly decreasing durations of anti-MRSA antibiotics with higher rates of early swabs (Wald test *P* value for deciles = .003 and spline terms <.001, eTable 7). In the model using restricted cubic splines, the association was not non-linear (Wald test *P* value for higher order spline terms = .12); thus, we performed a *post hoc* analysis considering swab percent use as a linear exposure which yielded similar results (eFigure 3B). Results were also robust across three of the four sensitivity analyses adjusting the cohort: excluding patients receiving dialysis (SHR for antibiotic discontinuation [95% CI]: 1.18 [1.05–1.33], *P* = .007); excluding patients receiving dialysis or with acute renal dysfunction (SHR [95% CI]: 1.24 [1.08–1.41], *P* = .002); and including all patients receiving early MV (irrespective of sepsis, SHR [95% CI]: 1.23 [1.07–1.41], *P* = .003; eFigures 4 A, B, and D; eTable 8). In all early sepsis patients (regardless of MV use), no association was observed (SHR [95% CI]: 1.05 [.95–1.16], *P* = .31; eFigure 4C).

Finally, in our “negative outcome control” analysis, the first duration of piperacillin-tazobactam and/or cefepime was also shorter among patients cared for in hospitals where ≥90% received an early MRSA nasal swab (median [IQR]: 3 [1, 6] vs 4 [2, 7] days, adjusted sub-hazard ratio [SHR] for antibiotic discontinuation [95% CI]: 1.16 [1.01–1.34], *P* = .035; eFigure 5, eTable 9).

## Discussion

In this large US multicenter cohort study of critically ill patients hospitalized with sepsis and requiring MV early in their course, we found use of early PCR-based MRSA nasal swabs varied substantially across hospitals and increased modestly from 2008 through 2021. Moreover, we found that patients cared for in hospitals that used early PCR-based MRSA nasal swabs and who received anti-MRSA antibiotics did so for shorter durations, suggesting evaluating for MRSA colonization may allow for earlier discontinuation. Being cared for in a hospital with higher rates of early MRSA nasal swab use was also associated with a lower hazard of new dialysis after hospital day 2 and, while statistical significance was not reached, possibly shorter durations of MV and lower in-hospital mortality. However, higher MRSA nasal swab use was also associated with shorter durations of non-anti-MRSA antibiotics (piperacillin-tazobactam and/or cefepime), suggesting frequent swab use may be a proxy for greater “antibiotic stewardship.”

The variability in use of early PCR-based MRSA swabs across hospitals is in line with prior studies of critically ill patients that show care is not standardized across sites.^20–22^ Surprisingly, we found that 22.5% of hospitals appeared to never swab a single eligible patient early in their hospital stay. This may reflect true non-use at the hospital level—an explanation supported by the decreasing frequency of this practice over time—or that these hospitals do, in fact, evaluate patients for colonization, but testing is not charged to the patient. For example, with universal screening programs, screening costs may be borne by the hospital.^23,24^ In an effort to better categorize the zero use hospitals, we investigated whether patients in them had outcomes more akin to those in <10% or ≥90% use hospitals, yet results were inconsistent across outcomes (data not shown). Therefore, our findings may underestimate the actual amount of early MRSA nasal swab-based colonization evaluation across the United States.

Importantly, our results suggest early PCR-based MRSA swabs may help to “rule-out” MRSA infection resulting in discontinuation of anti-MRSA antibiotics. This observation is in line with much of the existing literature in non-critically ill patients,^8–12,14,25^ although two studies found no such benefit.^15,16^ Reducing the use of anti-MRSA antibiotics has clear population-level benefits in mitigating against the emergence and propagation of new drug-resistant organisms (eg, vancomycin-intermediate *S. aureus*).^26,27^ Our findings suggest there may be individual patient benefit as well. Specifically, we found the hazard of renal failure leading to new dialysis was lower for patients in hospitals with higher rates of early MRSA colonization evaluation. Anti-MRSA antibiotics, specifically vancomycin, can cause acute kidney injury.^28^ Therefore, there is a plausible causal pathway in which higher rates of MRSA colonization evaluation may reduce anti-MRSA antibiotic durations which, in turn, may reduce rates of renal failure requiring new dialysis.

**Figure 4.**
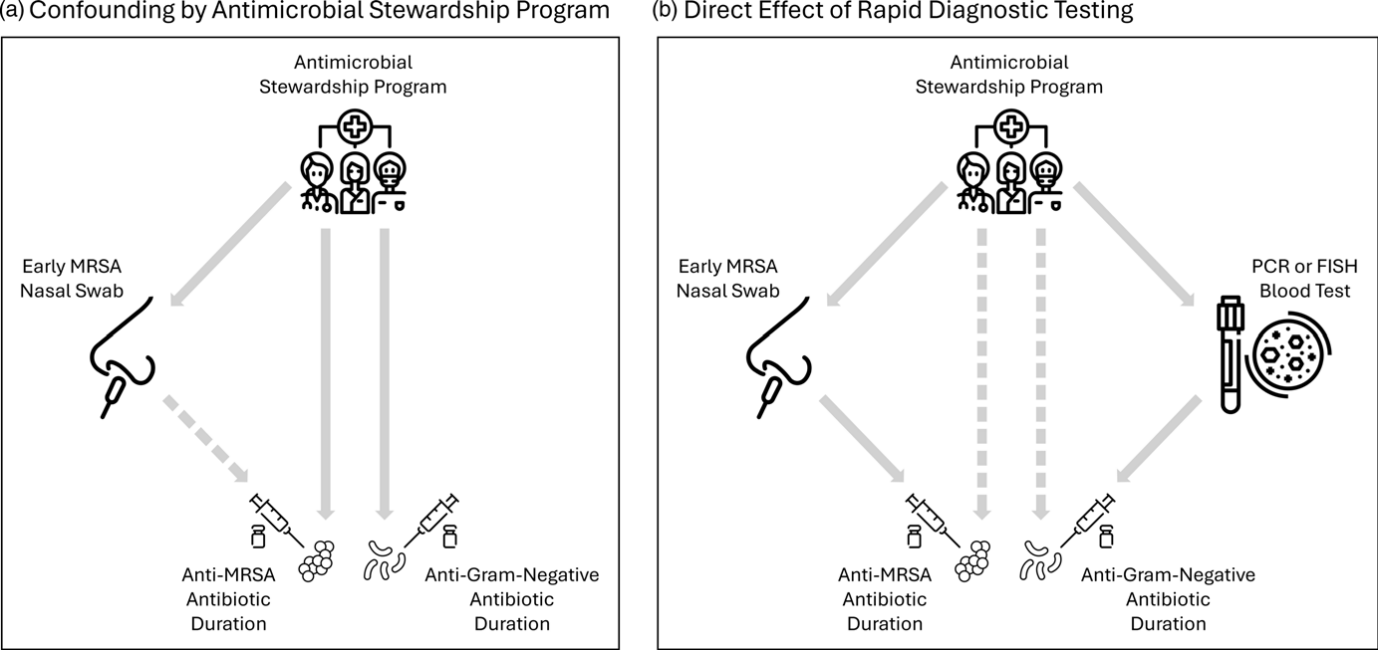
Framework for interpreting our “Negative outcome analysis” Findings.^*a*^ (A) Confounding by antimicrobial stewardship program. (B) Direct effect of rapid diagnostic testing. FISH, fluorescence in site hybridization; MRSA, methicillin-resistant *staphylococcus aureus*; PCR, polymerase chain reaction; *a* Solid gray arrows indicate definite causal links; dashed gray arrows indicate possible causal links. The scenarios depicted in panels A and B are meant to be illustrative of possibilities for how antimicrobial stewardship programs and early MRSA nasal swab testing may work together or separately to impact antibiotic durations; they are not meant to fully describe all possible scenarios. Moreover, it is possible that both the scenarios depicted in panels A and B may be at play simultaneously in a given setting.

Our “negative control outcome” analysis suggests that hospitals that use MRSA swabs may also be more attuned in other ways to reassessment of antibiotics in general. Specifically, antimicrobial stewardship programs are known to reduce antibiotic use,^29^ and a key component of such programs can be rapid diagnostic testing (eg, MRSA nasal swabs). In fact, antimicrobial stewardship programs combined with rapid testing has been shown to outperform stewardship programs alone.^30^ Therefore, it is possible that cohort hospitals with high early MRSA nasal swab use also have strong antimicrobial stewardship programs and these may reduce antibiotic use (anti-MRSA and not), unrelated to nasal testing (eg, through tracking and education^31^; Figure 4A). Another possibility, though, is that cohort hospitals with high MRSA swab use also have high use of other rapid diagnostic tests (eg, nucleic acid testing or fluorescence in site hybridization for g organism detection and susceptibility identification^32^; Figure 4B). Such testing allows for de-escalation of needlessly broad antibiotics (eg, piperacillin-tazobactam and/or cefepime).^33,34^ In this case the high use of early MRSA nasal swabs could be driving our observed reduction in anti-MRSA antibiotic duration, while a similarly high use of gram-negative bacteria rapid testing separately reduces piperacillin-tazobactam/cefepime duration.

The strengths of our study stem from its novelty in considering a critically ill population and its use of a large cohort from across hundreds of US hospitals. It also has limitations. First, as noted previously, there is concern about missing PCR-based MRSA nasal swabs which were performed but not charged to patients; this would result in misclassification of the outcome for the trends analyses and exposure for the outcomes analyses. Similarly, we only captured nasal swabs that used polymerase chain reaction tests; thus, we may have missed MRSA colonization evaluation done using culture. Second, we do not know why MRSA nasal swabs were performed (eg, for infection control surveillance or for antibiotic de-escalation). Third, we cannot know why anti-MRSA antibiotics were discontinued; such decisions may or may not have been related to MRSA nasal swab results. Fourth, we did not have access to MRSA nasal swab results and, therefore, could not investigate the differential impact of colonization evaluation on those who did versus did not rule out for MRSA colonization. Fifth, our findings may be impacted by residual confounding. Finally, while we used a large multicenter data set inclusive of hospitals across the US, the generalizability of our findings to other settings is unknown.

## Conclusions

Between 2008 and 2021, early PCR-based MRSA nasal swab use rose, but remained variable across US hospitals for critically ill patients with sepsis and respiratory failure. And, importantly, patients cared for in higher use hospitals had shorter durations of anti-MRSA antibiotics and better downstream outcomes. The cost-effectiveness of universal screening programs across all patient populations is unclear, especially as different approaches to isolation and decolonization abound.^35–40^ Yet, our findings suggest universal early MRSA nasal swab testing of critically ill patients with sepsis and respiratory failure may be effective in minimizing exposure to anti-MRSA treatment and its side effects. Further study is needed to understand the cost implications.

## Supporting information

10.1017/ash.2025.10093.sm001 Gershengorn et al. supplementary material Gershengorn et al. supplementary material

## Supplementary material

The supplementary material for this article can be found at https://doi.org/10.1017/ash.2025.10093
