# Supplementary material for: Methicillin-resistant Staphylococcus aureus nasal swabs: trends in use and association with outcomes
Source: Antimicrob Steward Healthc Epidemiol. 2025 Aug 7;5(1):e177. doi: 10.1017/ash.2025.10093 (PMC12345054; doi:10.1017/ash.2025.10093)
Supplement: Gershengorn et al. supplementary material [file S2732494X25100934sup001.docx]

**Methicillin-Resistant *Staphylococcus aureus* Nasal Swabs: Trends in Use and Association with Outcomes**

*Online Supplemental Material*

eTable 1. Patient- and Hospital-Related Model Covariables.

| Covariable | Definition | Categorization, if applicable |
| --- | --- | --- |
| Patient-Related |  |  |
| Age | As per PINC-AI |  |
| Gender*^a^* | As per PINC-AI | Male / Female |
| Race | As per PINC-AI | White / Black / Other |
| Hispanic ethnicity | As per PINC-AI | Yes / No |
| Primary payor | As per PINC-AI | Private / Medicare / Medicaid / Other |
| Individual Elixhauser comorbidities | By ICD-10 codes^1,2^ | Yes / No for each |
| Major surgery during the hospitalization | By Healthcare Cost and Utilization Project criteria^3^ | Yes / No |
| Receipt of vasopressors by hospital day 2 | As per PINC-AI charge codes | Yes / No |
| Receipt of dialysis by hospital day 2 | As per PINC-AI charge codes | Yes / No |
| Individual organ dysfunctions | By ICD-10 codes^4-6^ | Yes / No for each |
| Discharge quarter | As per PINC-AI | Individual quarter-years |
|  |  |  |
| Hospital-Related |  |  |
| Teaching hospital | As per PINC-AI |  |
| Number of hospital beds | As per PINC-AI | 500+ / 400-499 / 300-399 / 200-299 / 100-199 / 0-99 |
| U.S. Region | As per PINC-AI | Midwest / Northeast / South / West |
| Hospital environment | As per PINC-AI | Urban / Not urban |

ICD-10: International Classification of Diseases 10^th^ revision; PINC A1: PINC-A1 Healthcare Database

*a* PINC-AI uses the term “gender” rather than “sex”; thus, we have used the same throughout

*References*

1. Elixhauser A, Steiner C, Harris DR, Coffey RM. Comorbidity measures for use with administrative data. *Med Care*. Jan 1998;36(1):8-27.

2. Elixhauser Comorbidity Software for ICD-10-CM (beta version). Healthcare Cost and Utilization Project (HCUP). Agency for Healthcare Research and Quality. Accessed June 11, 2020. [www.hcup-us.ahrq.gov/toolssoftware/comorbidityicd10/comorbidity_icd10.jsp](file:///C:\Users\Owner\Box\__Research\MRSA%20swab%20epi%20and%20impact\www.hcup-us.ahrq.gov\toolssoftware\comorbidityicd10\comorbidity_icd10.jsp)

3. Healthcare Cost and Utilization Project: Beta Procedure Classes for ICD-10-PCS. Accessed September 25, 2019. <https://www.hcup-us.ahrq.gov/toolssoftware/procedureicd10/procedure_icd10.jsp>

4. Angus DC, Linde-Zwirble WT, Lidicker J, Clermont G, Carcillo J, Pinsky MR. Epidemiology of severe sepsis in the United States: analysis of incidence, outcome, and associated costs of care. *Crit Care Med*. Jul 2001;29(7):1303-10. doi:10.1097/00003246-200107000-00002

5. Carlton EF, Barbaro RP, Iwashyna TJ, Prescott HC. Cost of Pediatric Severe Sepsis Hospitalizations. *JAMA Pediatr*. Oct 01 2019;173(10):986-987. doi:10.1001/jamapediatrics.2019.2570

6. Prescott HC, Cope TM, Gesten FC, et al. Reporting of Sepsis Cases for Performance Measurement Versus for Reimbursement in New York State. *Crit Care Med*. May 2018;46(5):666-673. doi:10.1097/CCM.0000000000003005

eFigure 1. Flow Diagram.


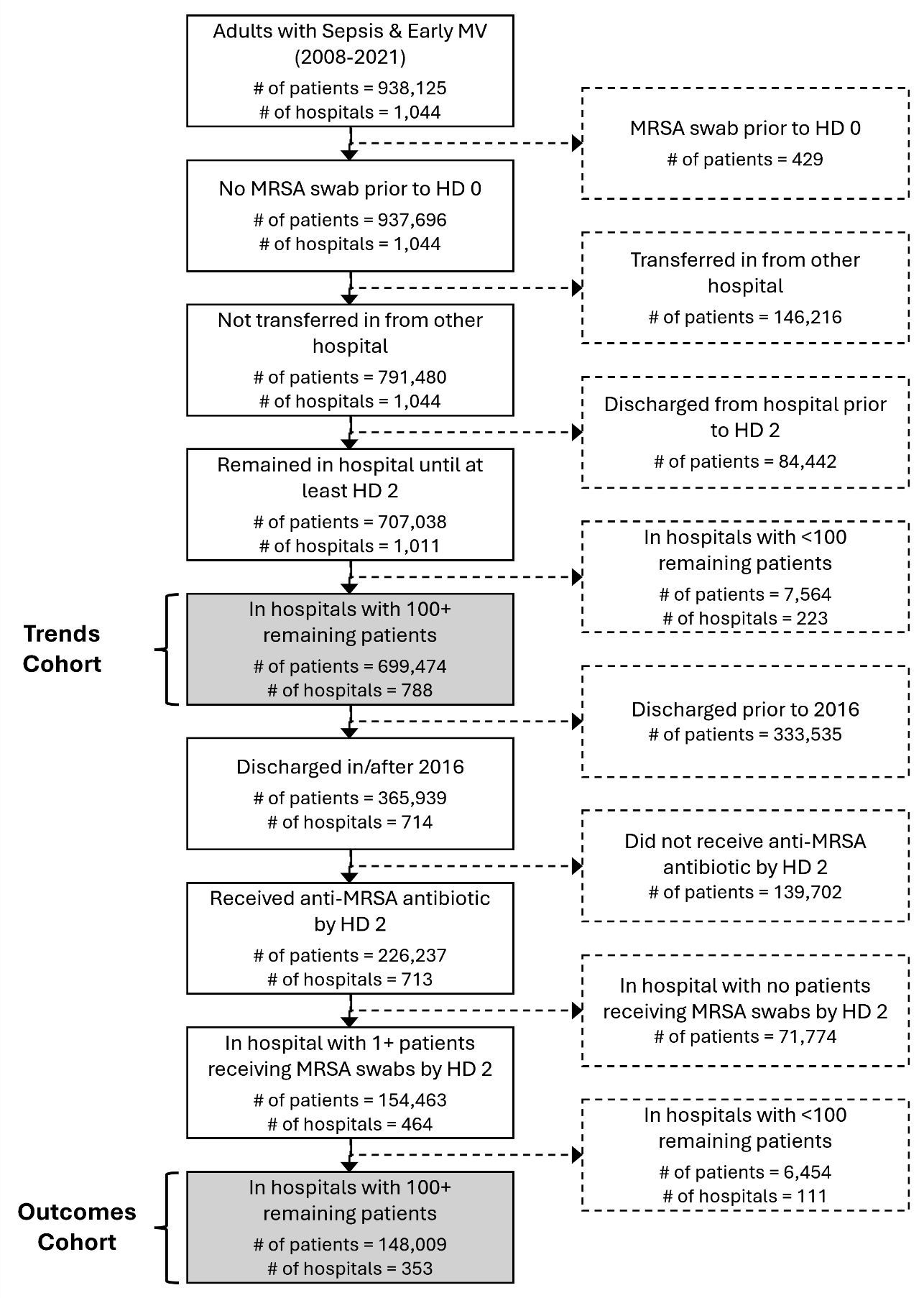


HD: hospital day; MRSA: methicillin-resistant *Staphylococcus aureus*; MV: mechanical ventilation

eTable 2. Individual Elixhauser Comorbidities for Cohort Evaluating Trends in Early MRSA Nasal Swab Use.

|  | no MRSA swab, N(%) | MRSA swab, N(%) | SMD |
| --- | --- | --- | --- |
| # of patients, N (row%) | 548,269 (78.4) | 151,205 (21.6) |  |
|  |  |  |  |
| Congestive heart failure | 222,826 (40.6) | 61,8101 (40.9) | 0.005 |
| Valvular disease | 61,367 (11.2) | 17,1441 (11.3) | 0.005 |
| Arrhythmia | 238,076 (43.4) | 66,1791 (43.8) | 0.007 |
| Hypertension, uncomplicated | 193,875 (35.4) | 50,9641 (33.7) | 0.035 |
| Hypertension, complicated | 181,678 (33.1) | 53,8391 (35.6) | 0.052 |
| Peripheral vascular disease | 61,494 (11.2) | 16,3681 (10.8) | 0.012 |
| Pulmonary disease | 269,140 (49.1) | 73,2081 (48.4) | 0.013 |
| Pulmonary circulatory disorders | 67,213 (12.3) | 20,0731 (13.3) | 0.030 |
| Diabetes mellitus, uncomplicated | 120,299 (21.9) | 29,0701 (19.2) | 0.067 |
| Diabetes mellitus, complicated | 107,521 (19.6) | 34,3851 (22.7) | 0.077 |
| Hypothyroidism | 83,442 (15.2) | 23,5571 (15.6) | 0.010 |
| Rheumatoid arthritis / collagen vascular disease | 20,203 (3.7) | 5,5311 (3.7) | 0.001 |
| AIDS/HIV | 4,094 (0.7) | 1,1551 (0.8) | 0.002 |
| Lymphoma | 5,599 (1.0) | 1,6121 (1.1) | 0.004 |
| Metastatic cancer | 16,831 (3.1) | 4,7271 (3.1) | 0.003 |
| Tumor (no metastases) | 31,677 (5.8) | 8,9491 (5.9) | 0.006 |
| Renal failure | 153,675 (28.0) | 43,3341 (28.7) | 0.014 |
| Liver disease | 77,486 (14.1) | 22,2801 (14.7) | 0.017 |
| Peptic ulcer disease | 6,751 (1.2) | 1,7651 (1.2) | 0.006 |
| Paralysis | 32,113 (5.9) | 9,2291 (6.1) | 0.010 |
| Other neurologic disorder | 243,842 (44.5) | 70,7971 (46.8) | 0.047 |
| Blood loss anemia | 8,653 (1.6) | 2,1451 (1.4) | 0.013 |
| Deficiency anemia | 33,796 (6.2) | 10,4111 (6.9) | 0.029 |
| Coagulopathy | 106,349 (19.4) | 30,2971 (20.0) | 0.016 |
| Obesity | 124,605 (22.7) | 35,3451 (23.4) | 0.015 |
| Weight loss | 113,502 (20.7) | 28,8651 (19.1) | 0.040 |
| Electrolyte disorder | 401,647 (73.3) | 112,9751 (74.7) | 0.033 |
| Depression | 93,879 (17.1) | 26,0231 (17.2) | 0.002 |
| Psychoses | 22,268 (4.1) | 6,5891 (4.4) | 0.015 |
| Alcohol abuse | 55,708 (10.2) | 15,2401 (10.1) | 0.003 |
| Drug abuse | 56,485 (10.3) | 16,5851 (11.0) | 0.022 |

MRSA: methicillin-resistant *Staphylococcus aureus*; SMD: standardized mean difference

eTable 3. Characteristics for Cohort Evaluating Trends in Early MRSA Nasal Swab Use, Stratified by Hospital-Level Early MRSA Nasal Swab Use.

|  | In Hospitals with 1+ Early MRSA Nasal Swab, N (%) | In Hospitals with Zero Early MRSA Nasal Swabs, N(%) | SMD |
| --- | --- | --- | --- |
| # of patients, N (row%) | 462,200 (66.1) | 237,274 (33.9) |  |
|  |  |  |  |
| Age, mean (sd) | 63.4 (15.5) | 63.9 (15.6) | 0.034 |
| Female gender | 224,829 (48.6) | 115,9911 (48.9) | 0.005 |
| Race |  |  | 0.145 |
| White | 330,656 (71.5) | 154,2761 (65.0) |  |
| Black | 70,939 (15.3) | 47,3171 (19.9) |  |
| Other/unknown | 60,605 (13.1) | 35,6811 (15.0) |  |
| Payor |  |  | 0.035 |
| Private | 62,448 (13.5) | 31,5891 (13.3) |  |
| Medicare | 289,332 (62.6) | 148,9331 (62.8) |  |
| Medicaid | 75,933 (16.4) | 40,8711 (17.2) |  |
| Other | 34,487 (7.5) | 15,8811 (6.7) |  |
| Elixhauser comorbidity # | 5.9 (2.4) | 6.0 (2.5) | 0.040 |
| Major surgery | 84,500 (18.3) | 41,3061 (17.4) | 0.023 |
| Resource use |  |  |  |
| Vasopressors by HD 2 | 206,935 (44.8) | 108,4681 (45.7) | 0.019 |
| Dialysis by HD 2 | 31,080 (6.7) | 17,3731 (7.3) | 0.023 |
| Acute organ dysfunctions |  |  |  |
| Renal | 221,112 (47.8) | 113,0091 (47.6) | 0.004 |
| Neurologic | 163,579 (35.4) | 85,6511 (36.1) | 0.015 |
| Liver | 30,035 (6.5) | 15,6781 (6.6) | 0.004 |
| Hematologic | 83,876 (18.1) | 43,6461 (18.4) | 0.006 |
| Cardiovascular | 228,257 (49.4) | 114,0521 (48.1) | 0.026 |
| Discharge year |  |  | 0.217 |
| 2008 | 10,780 (2.3) | 5,7201 (2.4) |  |
| 2009 | 15,479 (3.3) | 9,7951 (4.1) |  |
| 2010 | 19,439 (4.2) | 12,7061 (5.4) |  |
| 2011 | 26,090 (5.6) | 16,9701 (7.2) |  |
| 2012 | 29,065 (6.3) | 18,5711 (7.8) |  |
| 2013 | 32,640 (7.1) | 20,9881 (8.8) |  |
| 2014 | 34,822 (7.5) | 21,8621 (9.2) |  |
| 2015 | 36,424 (7.9) | 22,1841 (9.3) |  |
| 2016 | 39,454 (8.5) | 20,8791 (8.8) |  |
| 2017 | 46,492 (10.1) | 19,3241 (8.1) |  |
| 2018 | 45,614 (9.9) | 17,6661 (7.4) |  |
| 2019 | 44,730 (9.7) | 17,8881 (7.5) |  |
| 2020 | 44,600 (9.6) | 18,3491 (7.7) |  |
| 2021 | 36,571 (7.9) | 14,3721 (6.1) |  |
| Teaching hospital | 223,899 (48.4) | 122,9001 (51.8) | 0.067 |
| Hospital bed # |  |  | 0.129 |
| 500+ | 160,114 (34.6) | 79,5441 (33.5) |  |
| 400-499 | 66,297 (14.3) | 26,7251 (11.3) |  |
| 300-399 | 82,797 (17.9) | 50,8791 (21.4) |  |
| 200-299 | 83,659 (18.1) | 46,6731 (19.7) |  |
| 100-199 | 57,753 (12.5) | 28,6711 (12.1) |  |
| 0-99 | 11,580 (2.5) | 4,7821 (2.0) |  |
| Hospital region |  |  | 0.286 |
| Midwest | 81,187 (17.6) | 63,5181 (26.8) |  |
| Northeast | 69,295 (15.0) | 32,6691 (13.8) |  |
| South | 235,032 (50.9) | 92,2141 (38.9) |  |
| West | 76,686 (16.6) | 48,8731 (20.6) |  |
| Urban hospital | 412,306 (89.2) | 220,1291 (92.8) | 0.125 |

HD: hospital day; MRSA: methicillin-resistant *Staphylococcus aureus*; sd: standard deviation; SMD: standardized mean difference

eTable 4. Individual Elixhauser Comorbidities for Cohort Evaluating Trends in Early MRSA Nasal Swab Use, Stratified by Hospital-Level Early MRSA Nasal Swab Use.

|  | In Hospitals with 1+ Early MRSA Nasal Swab, N (%) | In Hospitals with Zero Early MRSA Nasal Swabs, N(%) | SMD |
| --- | --- | --- | --- |
| # of patients, N (row%) | 462,200 (66.1) | 237,274 (33.9) |  |
|  |  |  |  |
| Congestive heart failure | 186,907 (40.4) | 97,7291 (41.2) | 0.015 |
| Valvular disease | 50,619 (11.0) | 27,8921 (11.8) | 0.025 |
| Arrhythmia | 199,623 (43.2) | 104,6321 (44.1) | 0.018 |
| Hypertension, uncomplicated | 160,220 (34.7) | 84,6191 (35.7) | 0.021 |
| Hypertension, complicated | 155,699 (33.7) | 79,8181 (33.6) | <0.001 |
| Peripheral vascular disease | 50,607 (10.9) | 27,2551 (11.5) | 0.017 |
| Pulmonary disease | 225,095 (48.7) | 117,2531 (49.4) | 0.014 |
| Pulmonary circulatory disorders | 57,097 (12.4) | 30,1891 (12.7) | 0.011 |
| Diabetes mellitus, uncomplicated | 95,459 (20.7) | 53,9101 (22.7) | 0.050 |
| Diabetes mellitus, complicated | 96,133 (20.8) | 45,7731 (19.3) | 0.038 |
| Hypothyroidism | 70,404 (15.2) | 36,5951 (15.4) | 0.005 |
| Rheumatoid arthritis / collagen vascular disease | 16,881 (3.7) | 8,8531 (3.7) | 0.004 |
| AIDS/HIV | 3,346 (0.7) | 1,9031 (0.8) | 0.009 |
| Lymphoma | 4,800 (1.0) | 2,4111 (1.0) | 0.002 |
| Metastatic cancer | 14,098 (3.1) | 7,4601 (3.1) | 0.005 |
| Tumor (no metastases) | 26,855 (5.8) | 13,7711 (5.8) | <0.001 |
| Renal failure | 128,009 (27.7) | 69,0001 (29.1) | 0.031 |
| Liver disease | 66,060 (14.3) | 33,7061 (14.2) | 0.002 |
| Peptic ulcer disease | 5,428 (1.2) | 3,0881 (1.3) | 0.011 |
| Paralysis | 27,067 (5.9) | 14,2751 (6.0) | 0.007 |
| Other neurologic disorder | 206,123 (44.6) | 108,5161 (45.7) | 0.023 |
| Blood loss anemia | 6,643 (1.4) | 4,1551 (1.8) | 0.025 |
| Deficiency anemia | 29,325 (6.3) | 14,8821 (6.3) | 0.003 |
| Coagulopathy | 90,096 (19.5) | 46,5501 (19.6) | 0.003 |
| Obesity | 106,364 (23.0) | 53,5861 (22.6) | 0.010 |
| Weight loss | 89,675 (19.4) | 52,6921 (22.2) | 0.069 |
| Electrolyte disorder | 339,171 (73.4) | 175,4511 (73.9) | 0.013 |
| Depression | 80,959 (17.5) | 38,9431 (16.4) | 0.029 |
| Psychoses | 18,702 (4.0) | 10,1551 (4.3) | 0.012 |
| Alcohol abuse | 47,695 (10.3) | 23,2531 (9.8) | 0.017 |
| Drug abuse | 49,533 (10.7) | 23,5371 (9.9) | 0.026 |

MRSA: methicillin-resistant *Staphylococcus aureus*; SMD: standardized mean difference

eTable 5. Individual Elixhauser Comorbidities for Cohort Evaluating Association of Early MRSA Nasal Swab Use with Outcomes.

|  | Full Cohort, N(%) | In Hospitals with <10% Early MRSA Nasal Swabs, N(%) | In Hospitals with ≥90% Early MRSA Nasal Swabs, N(%) | SMD*^a^* |
| --- | --- | --- | --- | --- |
| # of patients, N (row%) | 41,559 (100.0) | 31,763 (76.4) | 9,796 (23.6) |  |
|  |  |  |  |  |
| Congestive heart failure | 18,255 (43.9) | 13,920 (43.8) | 4,3351 (44.3) | 0.009 |
| Valvular disease | 5,030 (12.1) | 3,630 (11.4) | 1,4001 (14.3) | 0.086 |
| Arrhythmia | 18,496 (44.5) | 14,041 (44.2) | 4,4551 (45.5) | 0.026 |
| Hypertension, uncomplicated | 11,436 (27.5) | 8,710 (27.4) | 2,7261 (27.8) | 0.009 |
| Hypertension, complicated | 18,475 (44.5) | 14,122 (44.5) | 4,3531 (44.4) | <0.001 |
| Peripheral vascular disease | 4,751 (11.4) | 3,657 (11.5) | 1,0941 (11.2) | 0.011 |
| Pulmonary disease | 18,370 (44.2) | 13,864 (43.6) | 4,5061 (46.0) | 0.047 |
| Pulmonary circulatory disorders | 5,807 (14.0) | 4,231 (13.3) | 1,5761 (16.1) | 0.078 |
| Diabetes mellitus, uncomplicated | 5,354 (12.9) | 4,149 (13.1) | 1,2051 (12.3) | 0.023 |
| Diabetes mellitus, complicated | 13,651 (32.8) | 10,514 (33.1) | 3,1371 (32.0) | 0.023 |
| Hypothyroidism | 6,919 (16.6) | 5,183 (16.3) | 1,7361 (17.7) | 0.037 |
| Rheumatoid arthritis / collagen vascular disease | 1,654 (4.0) | 1,294 (4.1) | 3601 (3.7) | 0.021 |
| AIDS/HIV | 378 (0.9) | 323 (1.0) | 551 (0.6) | 0.051 |
| Lymphoma | 511 (1.2) | 382 (1.2) | 1291 (1.3) | 0.010 |
| Metastatic cancer | 1,511 (3.6) | 1,125 (3.5) | 3861 (3.9) | 0.021 |
| Tumor (no metastases) | 2,784 (6.7) | 2,037 (6.4) | 7471 (7.6) | 0.047 |
| Renal failure | 13,104 (31.5) | 9,921 (31.2) | 3,1831 (32.5) | 0.027 |
| Liver disease | 7,453 (17.9) | 5,738 (18.1) | 1,7151 (17.5) | 0.015 |
| Peptic ulcer disease | 464 (1.1) | 343 (1.1) | 1211 (1.2) | 0.015 |
| Paralysis | 2,336 (5.6) | 1,791 (5.6) | 5451 (5.6) | 0.003 |
| Other neurologic disorder | 21,349 (51.4) | 16,524 (52.0) | 4,8251 (49.3) | 0.055 |
| Blood loss anemia | 575 (1.4) | 415 (1.3) | 1601 (1.6) | 0.027 |
| Deficiency anemia | 3,320 (8.0) | 2,423 (7.6) | 8971 (9.2) | 0.055 |
| Coagulopathy | 9,809 (23.6) | 7,423 (23.4) | 2,3861 (24.4) | 0.023 |
| Obesity | 10,215 (24.6) | 7,694 (24.2) | 2,5211 (25.7) | 0.035 |
| Weight loss | 8,847 (21.3) | 6,890 (21.7) | 1,9571 (20.0) | 0.042 |
| Electrolyte disorder | 33,383 (80.3) | 25,429 (80.1) | 7,9541 (81.2) | 0.029 |
| Depression | 7,205 (17.3) | 5,597 (17.6) | 1,6081 (16.4) | 0.032 |
| Psychoses | 1,556 (3.7) | 1,167 (3.7) | 3891 (4.0) | 0.015 |
| Alcohol abuse | 3,862 (9.3) | 2,930 (9.2) | 9321 (9.5) | 0.010 |
| Drug abuse | 4,953 (11.9) | 3,879 (12.2) | 1,0741 (11.0) | 0.039 |

MRSA: methicillin-resistant *Staphylococcus aureus*; SMD: standardized mean difference

*a* comparison of <10% versus ≥90% early MRSA nasal swab use hospitals

eFigure 2. Rates of Use of Anti-MRSA Antibiotics in the Outcomes Cohort Hospitals Stratified by Early MRSA Nasal Swab Use.*^a^*


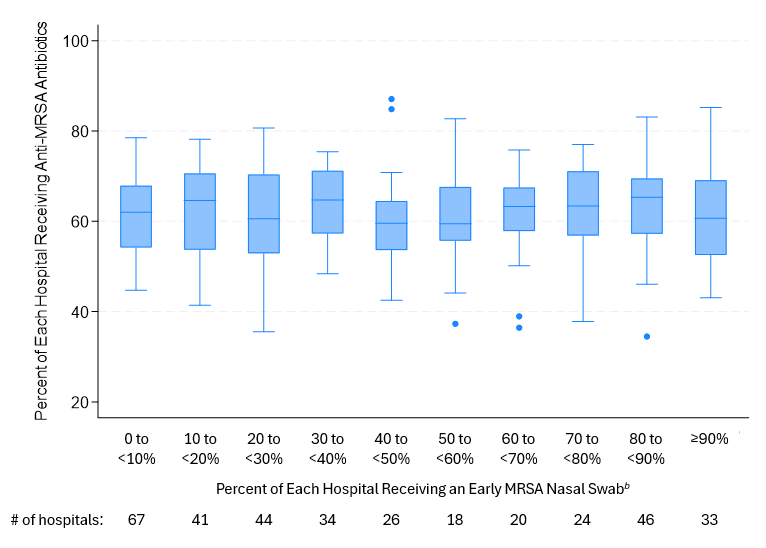


MRSA: methicillin-resistant *Staphylococcus aureus*

*a* Line = median of hospitals, blue box = interquartile range. Includes data from 238,108 patients in 353 hospitals since 2016, including 50,715 in 67 hospitals with <10% early MRSA nasal swab use and 15,966 in 33 hospitals with ≥90% swab use (the hospitals in our primary outcomes cohort). P-value for difference across all groups = 0.69; p-value for <10% vs ≥90% early swab use = 0.79.

*b* For consistency with outcomes analysis, the percentage of each hospital receiving an early MRSA swab is calculated from the outcomes cohort (i.e., just those patients started on anti-MRSA antibiotics by hospital day 2). However, there is high correlation between the rates of early MRSA nasal swabs among only patients receiving anti-MRSA antibiotics and all patients (Pearson correlation coefficient = 0.99).

eTable 6. Multivariable Time-to-Event Models.*^a^*

|  | Primary Outcome | | Secondary Outcomes | | | | | |
| --- | --- | --- | --- | --- | --- | --- | --- | --- |
|  | Discontinuation of Anti-MRSA Antibiotics | | Discontinuation of Mechanical Ventilation | | Hospital Mortality | | Initiation of Dialysis after Hospital Day 2*^b^* | |
|  | SHR (95% CI) | p-value | SHR (95% CI) | p-value | HR (95% CI) | p-value | SHR (95% CI) | p-value |
| In hospital with ≥90% (vs <10%) early MRSA nasal swab use | 1.17 (1.04,1.31) | 0.007 | 1.16 (0.98,1.37) | 0.08 | 0.88 (0.77,1.01) | 0.07 | 0.74 (0.61,0.89) | 0.001 |
|  |  |  |  |  |  |  |  |  |
| Age (per 1 year) | 1.00 (1.00,1.00) | <0.001 | 1.00 (1.00,1.00) | 0.62 | 1.01 (1.01,1.02) | <0.001 | 0.98 (0.97,0.98) | <0.001 |
| Female gender | 1.08 (1.06,1.10) | <0.001 | 0.99 (0.97,1.01) | 0.30 | 1.05 (1.01,1.10) | 0.027 | 0.85 (0.79,0.91) | <0.001 |
| Race |  |  |  |  |  |  |  |  |
| White | ref |  | ref |  | ref |  | ref |  |
| Black | 1.00 (0.95,1.05) | 0.95 | 0.88 (0.82,0.94) | <0.001 | 0.93 (0.87,1.00) | 0.07 | 1.23 (1.07,1.43) | 0.004 |
| Other/unknown | 0.99 (0.94,1.04) | 0.73 | 0.85 (0.77,0.93) | <0.001 | 1.01 (0.92,1.10) | 0.85 | 1.28 (1.12,1.45) | <0.001 |
| Hispanic ethnicity | 1.01 (0.96,1.07) | 0.66 | 0.96 (0.88,1.05) | 0.38 | 1.01 (0.92,1.12) | 0.80 | 1.22 (1.05,1.42) | 0.008 |
| Payor |  |  |  |  |  |  |  |  |
| Private | ref |  | ref |  | ref |  | ref |  |
| Medicare | 1.01 (0.98,1.04) | 0.40 | 1.00 (0.96,1.04) | 1.00 | 0.98 (0.91,1.05) | 0.63 | 0.93 (0.82,1.06) | 0.27 |
| Medicaid | 0.97 (0.94,1.01) | 0.11 | 0.95 (0.91,0.98) | 0.006 | 1.03 (0.94,1.12) | 0.57 | 0.78 (0.68,0.90) | 0.001 |
| Other | 1.04 (0.99,1.09) | 0.09 | 1.02 (0.96,1.08) | 0.61 | 1.18 (1.05,1.33) | 0.006 | 0.91 (0.74,1.12) | 0.39 |
| Elix. comorbidities |  |  |  |  |  |  |  |  |
| Congestive heart failure | 1.00 (0.98,1.03) | 0.69 | 1.04 (1.00,1.07) | 0.032 | 0.93 (0.89,0.98) | 0.005 | 1.03 (0.93,1.15) | 0.53 |
| Valvular disease | 0.98 (0.95,1.02) | 0.38 | 1.00 (0.96,1.04) | 0.88 | 1.02 (0.96,1.08) | 0.50 | 1.01 (0.90,1.14) | 0.84 |
| Arrhythmia | 0.98 (0.96,1.00) | 0.012 | 0.90 (0.87,0.92) | <0.001 | 1.22 (1.17,1.28) | <0.001 | 1.19 (1.09,1.30) | <0.001 |
| Hypertension, uncomplicated | 0.97 (0.94,1.00) | 0.044 | 1.07 (1.03,1.10) | <0.001 | 0.93 (0.88,0.99) | 0.015 | 1.11 (0.97,1.27) | 0.12 |
| Hypertension, complicated | 1.02 (0.99,1.05) | 0.28 | 1.10 (1.06,1.13) | <0.001 | 0.84 (0.80,0.89) | <0.001 | 1.09 (0.97,1.24) | 0.15 |
| Peripheral vascular disease | 1.00 (0.97,1.03) | 0.99 | 1.04 (1.01,1.08) | 0.019 | 1.13 (1.07,1.19) | <0.001 | 0.88 (0.79,0.97) | 0.009 |
| Pulmonary disease | 1.01 (0.99,1.03) | 0.25 | 1.02 (0.99,1.05) | 0.20 | 0.94 (0.90,0.99) | 0.013 | 0.81 (0.74,0.89) | <0.001 |
| Pulmonary circulatory disorders | 0.95 (0.92,0.98) | <0.001 | 0.93 (0.90,0.96) | <0.001 | 1.10 (1.04,1.17) | 0.001 | 1.09 (0.97,1.23) | 0.15 |
| Diabetes mellitus, uncomplicated | 1.01 (0.99,1.04) | 0.31 | 0.95 (0.93,0.98) | <0.001 | 0.99 (0.94,1.05) | 0.81 | 1.25 (1.08,1.45) | 0.002 |
| Diabetes mellitus, complicated | 0.97 (0.96,0.99) | 0.005 | 1.00 (0.97,1.03) | 0.92 | 0.95 (0.89,1.00) | 0.05 | 1.24 (1.13,1.36) | <0.001 |
| Hypothyroidism | 0.98 (0.96,1.00) | 0.028 | 1.03 (1.00,1.06) | 0.040 | 0.88 (0.84,0.93) | <0.001 | 1.01 (0.91,1.12) | 0.85 |
| Rheumatoid arthritis / collagen vascular disease | 0.99 (0.95,1.03) | 0.53 | 1.03 (0.98,1.08) | 0.19 | 1.01 (0.92,1.12) | 0.77 | 0.92 (0.76,1.11) | 0.40 |
| AIDS/HIV | 1.05 (0.95,1.17) | 0.32 | 1.01 (0.89,1.14) | 0.92 | 1.30 (1.07,1.57) | 0.008 | 0.96 (0.66,1.41) | 0.85 |
| Lymphoma | 0.94 (0.88,1.01) | 0.11 | 1.01 (0.92,1.10) | 0.90 | 1.21 (1.06,1.38) | 0.005 | 1.27 (0.96,1.68) | 0.09 |
| Metastatic cancer | 0.95 (0.90,1.01) | 0.13 | 0.92 (0.85,1.00) | 0.040 | 1.50 (1.35,1.65) | <0.001 | 0.75 (0.54,1.06) | 0.10 |
| Tumor (no metastases) | 1.01 (0.96,1.06) | 0.81 | 0.94 (0.89,1.00) | 0.05 | 1.41 (1.31,1.53) | <0.001 | 0.82 (0.68,0.99) | 0.042 |
| Renal failure | 1.19 (1.15,1.23) | <0.001 | 1.00 (0.97,1.03) | 0.98 | 1.11 (1.04,1.18) | 0.001 | 4.53 (3.91,5.26) | <0.001 |
| Liver disease | 1.02 (0.99,1.05) | 0.19 | 0.91 (0.88,0.95) | <0.001 | 1.35 (1.24,1.48) | <0.001 | 1.26 (1.11,1.43) | <0.001 |
| Peptic ulcer disease | 0.95 (0.89,1.02) | 0.18 | 1.04 (0.95,1.12) | 0.40 | 0.71 (0.57,0.87) | 0.001 | 0.98 (0.74,1.30) | 0.90 |
| Paralysis | 0.97 (0.93,1.01) | 0.19 | 0.92 (0.88,0.96) | <0.001 | 0.89 (0.82,0.98) | 0.013 | 0.79 (0.65,0.97) | 0.021 |
| Other neurologic disorder | 0.99 (0.96,1.02) | 0.34 | 0.94 (0.91,0.97) | <0.001 | 0.95 (0.88,1.03) | 0.21 | 0.81 (0.71,0.92) | 0.002 |
| Blood loss anemia | 0.97 (0.90,1.05) | 0.51 | 0.96 (0.90,1.03) | 0.31 | 0.88 (0.74,1.03) | 0.11 | 0.89 (0.67,1.17) | 0.40 |
| Deficiency anemia | 0.98 (0.94,1.01) | 0.22 | 1.03 (0.98,1.07) | 0.22 | 0.71 (0.65,0.77) | <0.001 | 1.00 (0.88,1.14) | 0.95 |
| Coagulopathy | 0.99 (0.93,1.05) | 0.68 | 0.91 (0.84,1.00) | 0.038 | 1.15 (0.99,1.33) | 0.07 | 1.48 (1.15,1.90) | 0.002 |
| Obesity | 0.97 (0.95,1.00) | 0.024 | 0.92 (0.89,0.96) | <0.001 | 0.93 (0.88,0.98) | 0.010 | 1.11 (1.02,1.21) | 0.015 |
| Weight loss | 0.95 (0.93,0.97) | <0.001 | 0.96 (0.92,1.00) | 0.036 | 0.88 (0.84,0.93) | <0.001 | 1.20 (1.09,1.33) | <0.001 |
| Electrolyte disorder | 0.97 (0.95,1.00) | 0.044 | 0.91 (0.88,0.95) | <0.001 | 1.13 (1.06,1.21) | <0.001 | 1.25 (1.07,1.47) | 0.006 |
| Depression | 0.98 (0.96,1.01) | 0.24 | 1.09 (1.05,1.12) | <0.001 | 0.82 (0.77,0.89) | <0.001 | 0.98 (0.88,1.08) | 0.63 |
| Psychoses | 0.95 (0.91,1.00) | 0.05 | 1.13 (1.08,1.19) | <0.001 | 0.65 (0.55,0.77) | <0.001 | 0.65 (0.50,0.85) | 0.002 |
| Alcohol abuse | 1.05 (1.01,1.09) | 0.008 | 1.02 (0.98,1.07) | 0.25 | 1.00 (0.92,1.08) | 0.90 | 0.93 (0.83,1.05) | 0.24 |
| Drug abuse | 0.95 (0.91,0.99) | 0.024 | 1.15 (1.10,1.21) | <0.001 | 0.74 (0.67,0.80) | <0.001 | 0.88 (0.76,1.02) | 0.08 |
| Major surgery | 0.94 (0.92,0.97) | <0.001 | 1.01 (0.97,1.05) | 0.61 | 0.59 (0.55,0.63) | <0.001 | 1.62 (1.47,1.78) | <0.001 |
| Resource use |  |  |  |  |  |  |  |  |
| Vasopressors by HD 2 | 1.00 (0.97,1.03) | 0.78 | 0.86 (0.82,0.90) | <0.001 | 1.60 (1.50,1.70) | <0.001 | 1.12 (1.02,1.24) | 0.022 |
| Dialysis by HD 2 | 1.33 (1.26,1.41) | <0.001 | 1.05 (0.99,1.11) | 0.12 | 1.15 (1.07,1.23) | <0.001 | omitted |  |
| Acute organ dysfunc |  |  |  |  |  |  |  |  |
| Renal | 1.08 (1.06,1.11) | <0.001 | 0.90 (0.87,0.93) | <0.001 | 1.36 (1.29,1.43) | <0.001 | 1.15 (0.95,1.39) | 0.158 |
| Neurologic | 1.02 (0.99,1.05) | 0.15 | 0.92 (0.89,0.96) | <0.001 | 1.24 (1.15,1.33) | <0.001 | 1.38 (1.20,1.59) | <0.001 |
| Liver | 1.02 (0.98,1.07) | 0.26 | 0.80 (0.76,0.85) | <0.001 | 1.40 (1.28,1.52) | <0.001 | 1.51 (1.30,1.77) | <0.001 |
| Hematologic | 1.02 (0.95,1.09) | 0.62 | 0.97 (0.89,1.05) | 0.41 | 1.04 (0.89,1.21) | 0.63 | 1.27 (1.00,1.63) | 0.05 |
| Cardiovascular | 0.94 (0.91,0.96) | <0.001 | 0.80 (0.77,0.83) | <0.001 | 1.73 (1.60,1.86) | <0.001 | 2.00 (1.76,2.27) | <0.001 |
| Discharge year |  |  |  |  |  |  |  |  |
| 2016 | ref |  | ref |  | ref |  | ref |  |
| 2017 | 1.01 (0.96,1.06) | 0.69 | 0.99 (0.95,1.04) | 0.78 | 1.00 (0.91,1.10) | 0.99 | 0.95 (0.82,1.09) | 0.45 |
| 2018 | 1.03 (0.97,1.09) | 0.38 | 0.99 (0.93,1.05) | 0.75 | 1.00 (0.91,1.10) | 0.97 | 1.01 (0.87,1.18) | 0.88 |
| 2019 | 1.04 (0.98,1.10) | 0.21 | 1.00 (0.93,1.06) | 0.91 | 0.95 (0.85,1.06) | 0.34 | 1.08 (0.92,1.27) | 0.36 |
| 2020 | 1.05 (0.99,1.12) | 0.08 | 0.91 (0.84,1.00) | 0.045 | 1.15 (1.04,1.28) | 0.006 | 1.14 (0.97,1.36) | 0.12 |
| 2021 | 1.06 (1.00,1.13) | 0.048 | 0.88 (0.81,0.97) | 0.007 | 1.31 (1.19,1.44) | <0.001 | 0.97 (0.79,1.19) | 0.77 |
| Teaching hospital | 1.04 (0.94,1.16) | 0.45 | 0.80 (0.67,0.94) | 0.009 | 1.19 (1.01,1.41) | 0.041 | 1.06 (0.87,1.27) | 0.57 |
| Hospital bed # |  |  |  |  |  |  |  |  |
| 500+ | ref |  | ref |  | ref |  | ref |  |
| 400-499 | 1.06 (0.91,1.24) | 0.46 | 1.00 (0.82,1.22) | 1.00 | 1.20 (0.97,1.48) | 0.10 | 0.95 (0.69,1.31) | 0.75 |
| 300-399 | 0.91 (0.82,1.02) | 0.11 | 0.93 (0.80,1.09) | 0.37 | 1.06 (0.88,1.27) | 0.54 | 0.96 (0.77,1.20) | 0.70 |
| 200-299 | 1.05 (0.91,1.20) | 0.49 | 1.10 (0.92,1.31) | 0.31 | 1.25 (1.00,1.56) | 0.046 | 1.09 (0.87,1.37) | 0.45 |
| 100-199 | 1.02 (0.89,1.18) | 0.74 | 1.06 (0.86,1.31) | 0.60 | 0.97 (0.78,1.20) | 0.78 | 1.11 (0.81,1.54) | 0.51 |
| 0-99 | 1.10 (0.86,1.40) | 0.44 | 1.19 (0.92,1.54) | 0.18 | 0.96 (0.73,1.25) | 0.76 | 0.91 (0.50,1.68) | 0.77 |
| Hospital region |  |  |  |  |  |  |  |  |
| Midwest | ref |  | ref |  | ref |  | ref |  |
| Northeast | 1.38 (1.15,1.65) | <0.001 | 1.65 (1.21,2.25) | 0.002 | 1.17 (0.98,1.40) | 0.08 | 0.78 (0.52,1.16) | 0.22 |
| South | 1.14 (1.04,1.25) | 0.003 | 0.99 (0.89,1.10) | 0.89 | 1.07 (0.94,1.22) | 0.30 | 0.98 (0.73,1.31) | 0.88 |
| West | 1.03 (0.88,1.20) | 0.75 | 0.78 (0.61,0.99) | 0.043 | 1.34 (1.13,1.58) | 0.001 | 1.02 (0.75,1.38) | 0.90 |
| Urban hospital | 1.15 (0.96,1.38) | 0.13 | 1.04 (0.80,1.34) | 0.78 | 1.01 (0.87,1.18) | 0.88 | 1.01 (0.75,1.35) | 0.97 |

Elix.: Elixhauser; dysfunc.: dysfunction; HD: hospital day; HR: hazard ratio; MRSA: methicillin-resistant *Staphylococcus aureus*; ref: reference; SHR: sub-hazard ratio

*a* Using multivariable competing risks models (except mortality which is time-to-death)

*b* Restricted to patients who did not receive dialysis on or before hospital day 2

eFigure 3. Association of Hospital-Level Early MRSA Nasal Swab Use and Discontinuation of Anti-MRSA Antibiotics Using Sensitivity Exposures.*^a^*


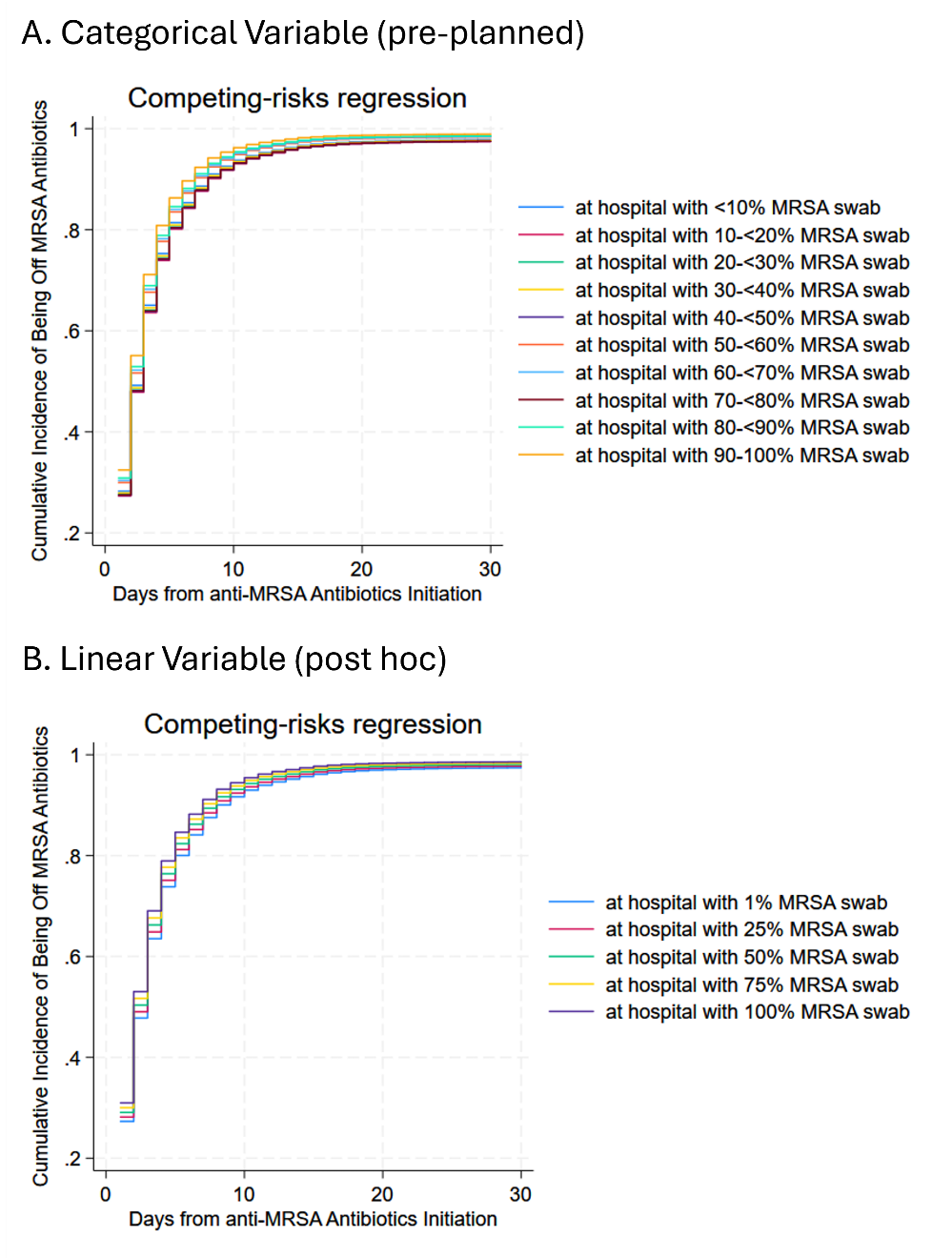


*a* Using multivariable competing risks models; Panel A: Wald test for categorical values, p=0.003; Panel B: p<0.001

eTable 7. Multivariable Time-to-Event Models Using Sensitivity Exposures.*^a^*

|  | Categorical Exposure | | Restricted Cubic Spline Exp.* | | Linear Exposure (post hoc) | |
| --- | --- | --- | --- | --- | --- | --- |
|  | SHR (95% CI) | p-value | SHR (95% CI) | p-value | SHR (95% CI) | p-value |
| % with MRSA swab on HD 0-2 |  |  |  |  |  |  |
| <10 | ref |  |  |  |  |  |
| 10-<20 | 0.96 (0.90,1.03) | 0.27 |  |  |  |  |
| 20-<30 | 0.97 (0.90,1.06) | 0.52 |  |  |  |  |
| 30-<40 | 0.99 (0.91,1.07) | 0.73 |  |  |  |  |
| 40-<50 | 0.97 (0.90,1.04) | 0.37 |  |  |  |  |
| 50-<60 | 1.07 (0.98,1.18) | 0.13 |  |  |  |  |
| 60-<70 | 1.09 (1.00,1.19) | 0.06 |  |  |  |  |
| 70-<80 | 0.97 (0.90,1.05) | 0.43 |  |  |  |  |
| 80-<90 | 1.11 (1.02,1.21) | 0.015 |  |  |  |  |
| 90-100 | 1.18 (1.04,1.34) | 0.009 |  |  |  |  |
|  |  |  |  |  |  |  |
| spline term 1 |  |  | 0.89 (0.57,1.39) | 0.61 |  |  |
| spline term 2 |  |  | 1.88 (0.20,17.91) | 0.58 |  |  |
| spline term 3 |  |  | 0.46 (0.00,46.82) | 0.74 |  |  |
|  |  |  |  |  |  |  |
| linear term (per 1% increase) |  |  |  |  | 1.002 (1.001,1.002) | <0.001 |
|  |  |  |  |  |  |  |
| Age (per 1 year) | 1.00 (1.00,1.00) | <0.001 | 1.00 (1.00,1.00) | <0.001 | 1.00 (1.00,1.00) | <0.001 |
| Female gender | 1.08 (1.07,1.09) | <0.001 | 1.08 (1.07,1.09) | <0.001 | 1.08 (1.07,1.09) | <0.001 |
| Race |  |  |  |  |  |  |
| White | ref |  | ref |  | ref |  |
| Black | 1.02 (1.00,1.04) | 0.09 | 1.02 (1.00,1.05) | 0.09 | 1.02 (1.00,1.05) | 0.06 |
| Other/unknown | 1.00 (0.97,1.03) | 0.85 | 1.00 (0.97,1.03) | 0.92 | 1.00 (0.97,1.03) | 0.99 |
| Hispanic ethnicity | 1.00 (0.97,1.03) | 0.93 | 0.99 (0.96,1.03) | 0.71 | 1.00 (0.97,1.03) | 0.90 |
| Payor |  |  |  |  |  |  |
| Private | ref |  | ref |  | ref |  |
| Medicare | 1.02 (1.00,1.03) | 0.06 | 1.02 (1.00,1.03) | 0.06 | 1.02 (1.00,1.03) | 0.07 |
| Medicaid | 0.99 (0.97,1.00) | 0.14 | 0.99 (0.97,1.01) | 0.16 | 0.99 (0.97,1.01) | 0.15 |
| Other | 1.03 (1.01,1.06) | 0.013 | 1.03 (1.01,1.06) | 0.017 | 1.03 (1.00,1.06) | 0.026 |
| Elixhauser comorbidities |  |  |  |  |  |  |
| Congestive heart failure | 1.01 (1.00,1.03) | 0.07 | 1.01 (1.00,1.03) | 0.06 | 1.01 (1.00,1.03) | 0.06 |
| Valvular disease | 0.99 (0.97,1.00) | 0.07 | 0.99 (0.97,1.00) | 0.11 | 0.99 (0.97,1.00) | 0.10 |
| Arrhythmia | 0.98 (0.97,0.99) | <0.001 | 0.98 (0.97,0.99) | <0.001 | 0.98 (0.97,0.99) | <0.001 |
| Hypertension, uncomplicated | 0.99 (0.98,1.01) | 0.40 | 1.00 (0.98,1.01) | 0.52 | 1.00 (0.98,1.01) | 0.52 |
| Hypertension, complicated | 1.03 (1.01,1.04) | 0.004 | 1.03 (1.01,1.04) | 0.003 | 1.03 (1.01,1.05) | 0.003 |
| Peripheral vascular disease | 1.01 (0.99,1.02) | 0.37 | 1.01 (0.99,1.02) | 0.42 | 1.01 (0.99,1.02) | 0.41 |
| Pulmonary disease | 1.00 (0.99,1.01) | 0.94 | 1.00 (0.99,1.01) | 0.99 | 1.00 (0.99,1.01) | 0.97 |
| Pulmonary circulatory disorders | 0.98 (0.96,0.99) | 0.004 | 0.98 (0.96,0.99) | 0.006 | 0.98 (0.96,0.99) | 0.009 |
| Diabetes mellitus, uncomplicated | 0.99 (0.98,1.01) | 0.27 | 0.99 (0.98,1.01) | 0.26 | 0.99 (0.98,1.01) | 0.25 |
| Diabetes mellitus, complicated | 0.98 (0.97,0.99) | <0.001 | 0.98 (0.97,0.99) | <0.001 | 0.98 (0.97,0.99) | <0.001 |
| Hypothyroidism | 0.99 (0.97,1.00) | 0.012 | 0.99 (0.98,1.00) | 0.026 | 0.99 (0.98,1.00) | 0.034 |
| Rheumatoid arthritis / collagen vascular disease | 0.97 (0.95,0.99) | 0.014 | 0.97 (0.95,1.00) | 0.016 | 0.97 (0.95,1.00) | 0.017 |
| AIDS/HIV | 1.05 (1.00,1.10) | 0.07 | 1.05 (1.00,1.10) | 0.07 | 1.05 (1.00,1.10) | 0.06 |
| Lymphoma | 0.95 (0.91,0.99) | 0.013 | 0.95 (0.91,0.99) | 0.012 | 0.95 (0.91,0.99) | 0.012 |
| Metastatic cancer | 0.96 (0.93,0.99) | 0.011 | 0.96 (0.93,0.99) | 0.009 | 0.96 (0.93,0.99) | 0.009 |
| Tumor (no metastases) | 0.97 (0.94,0.99) | 0.006 | 0.97 (0.94,0.99) | 0.008 | 0.97 (0.94,0.99) | 0.007 |
| Renal failure | 1.21 (1.19,1.23) | <0.001 | 1.21 (1.19,1.23) | <0.001 | 1.21 (1.19,1.23) | <0.001 |
| Liver disease | 1.02 (1.00,1.03) | 0.06 | 1.02 (1.00,1.03) | 0.05 | 1.02 (1.00,1.04) | 0.046 |
| Peptic ulcer disease | 0.99 (0.95,1.03) | 0.72 | 0.99 (0.95,1.03) | 0.71 | 0.99 (0.95,1.03) | 0.70 |
| Paralysis | 0.96 (0.94,0.97) | <0.001 | 0.96 (0.94,0.98) | <0.001 | 0.96 (0.94,0.97) | <0.001 |
| Other neurologic disorder | 0.98 (0.97,1.00) | 0.049 | 0.98 (0.97,1.00) | 0.05 | 0.98 (0.97,1.00) | 0.05 |
| Blood loss anemia | 0.97 (0.93,1.01) | 0.14 | 0.97 (0.93,1.01) | 0.17 | 0.97 (0.93,1.01) | 0.17 |
| Deficiency anemia | 0.98 (0.97,1.00) | 0.046 | 0.98 (0.97,1.00) | 0.046 | 0.98 (0.97,1.00) | 0.06 |
| Coagulopathy | 0.97 (0.93,1.02) | 0.23 | 0.97 (0.93,1.02) | 0.26 | 0.97 (0.93,1.01) | 0.16 |
| Obesity | 0.97 (0.96,0.98) | <0.001 | 0.97 (0.96,0.99) | <0.001 | 0.97 (0.96,0.99) | <0.001 |
| Weight loss | 0.93 (0.92,0.95) | <0.001 | 0.93 (0.92,0.95) | <0.001 | 0.94 (0.92,0.95) | <0.001 |
| Electrolyte disorder | 0.97 (0.96,0.99) | <0.001 | 0.97 (0.96,0.99) | <0.001 | 0.97 (0.96,0.99) | <0.001 |
| Depression | 0.99 (0.98,1.00) | 0.16 | 0.99 (0.98,1.01) | 0.26 | 0.99 (0.98,1.01) | 0.26 |
| Psychoses | 0.97 (0.95,0.99) | 0.006 | 0.97 (0.95,0.99) | 0.006 | 0.97 (0.95,0.99) | 0.005 |
| Alcohol abuse | 1.05 (1.03,1.07) | <0.001 | 1.05 (1.03,1.07) | <0.001 | 1.05 (1.03,1.07) | <0.001 |
| Drug abuse | 0.97 (0.95,0.99) | 0.002 | 0.97 (0.96,0.99) | 0.004 | 0.97 (0.96,0.99) | 0.003 |
| Major surgery | 0.92 (0.91,0.94) | <0.001 | 0.92 (0.91,0.94) | <0.001 | 0.92 (0.91,0.94) | <0.001 |
| Resource use |  |  |  |  |  |  |
| Vasopressors by HD 2 | 1.00 (0.98,1.01) | 0.64 | 1.00 (0.98,1.01) | 0.69 | 1.00 (0.98,1.01) | 0.65 |
| Dialysis by HD 2 | 1.25 (1.20,1.29) | <0.001 | 1.25 (1.20,1.29) | <0.001 | 1.24 (1.20,1.29) | <0.001 |
| Acute organ dysfunctions |  |  |  |  |  |  |
| Renal | 1.08 (1.07,1.10) | <0.001 | 1.08 (1.07,1.09) | <0.001 | 1.08 (1.07,1.10) | <0.001 |
| Neurologic | 1.01 (0.99,1.02) | 0.43 | 1.00 (0.99,1.02) | 0.56 | 1.00 (0.99,1.02) | 0.66 |
| Liver | 1.02 (0.99,1.04) | 0.14 | 1.02 (1.00,1.04) | 0.11 | 1.02 (0.99,1.04) | 0.13 |
| Hematologic | 1.03 (0.98,1.07) | 0.29 | 1.02 (0.98,1.07) | 0.32 | 1.03 (0.98,1.07) | 0.21 |
| Cardiovascular | 0.94 (0.92,0.95) | <0.001 | 0.94 (0.92,0.95) | <0.001 | 0.94 (0.92,0.95) | <0.001 |
| Discharge year |  |  |  |  |  |  |
| 2016 | ref |  | ref |  | ref |  |
| 2017 | 1.03 (1.01,1.06) | 0.008 | 1.03 (1.01,1.06) | 0.009 | 1.03 (1.01,1.06) | 0.007 |
| 2018 | 1.05 (1.02,1.08) | <0.001 | 1.05 (1.02,1.08) | 0.001 | 1.05 (1.02,1.08) | 0.001 |
| 2019 | 1.09 (1.06,1.13) | <0.001 | 1.09 (1.06,1.13) | <0.001 | 1.09 (1.06,1.13) | <0.001 |
| 2020 | 1.12 (1.08,1.16) | <0.001 | 1.12 (1.08,1.16) | <0.001 | 1.12 (1.08,1.16) | <0.001 |
| 2021 | 1.15 (1.11,1.19) | <0.001 | 1.15 (1.11,1.19) | <0.001 | 1.15 (1.11,1.19) | <0.001 |
| Teaching hospital | 1.03 (0.98,1.09) | 0.19 | 1.03 (0.98,1.09) | 0.20 | 1.03 (0.98,1.09) | 0.20 |
| Hospital bed # |  |  |  |  |  |  |
| 500+ | ref |  | ref |  | ref |  |
| 400-499 | 1.01 (0.94,1.08) | 0.84 | 1.01 (0.94,1.08) | 0.87 | 1.00 (0.94,1.07) | 0.94 |
| 300-399 | 0.93 (0.87,0.99) | 0.025 | 0.93 (0.87,0.99) | 0.035 | 0.93 (0.87,1.00) | 0.036 |
| 200-299 | 1.01 (0.94,1.07) | 0.87 | 1.00 (0.93,1.07) | 0.98 | 1.01 (0.94,1.08) | 0.82 |
| 100-199 | 0.97 (0.90,1.04) | 0.37 | 0.97 (0.90,1.04) | 0.35 | 0.97 (0.90,1.04) | 0.35 |
| 0-99 | 0.93 (0.80,1.07) | 0.30 | 0.93 (0.80,1.08) | 0.35 | 0.93 (0.80,1.08) | 0.35 |
| Hospital region |  |  |  |  |  |  |
| Midwest | ref |  | ref |  | ref |  |
| Northeast | 1.14 (1.05,1.25) | 0.003 | 1.13 (1.04,1.24) | 0.006 | 1.14 (1.04,1.25) | 0.005 |
| South | 0.99 (0.94,1.05) | 0.74 | 0.99 (0.94,1.05) | 0.79 | 1.00 (0.95,1.05) | 0.94 |
| West | 0.95 (0.89,1.02) | 0.14 | 0.94 (0.88,1.01) | 0.08 | 0.95 (0.90,1.02) | 0.15 |
| Urban hospital | 1.06 (0.99,1.14) | 0.10 | 1.06 (0.99,1.14) | 0.11 | 1.06 (0.99,1.14) | 0.09 |

Exp.: Exposure; HD: hospital day; MRSA: methicillin-resistant *Staphylococcus aureus*; ref: reference; SHR: sub-hazard ratio

*a* Using multivariable competing risks models; Wald test for exposure components for categorical exposure p=0.003 and spline terms p<0.001

eFigure 4. Association of Hospital-Level Early MRSA Nasal Swab Use and Discontinuation of Anti-MRSA Antibiotics Using Sensitivity Cohorts.*^a^*


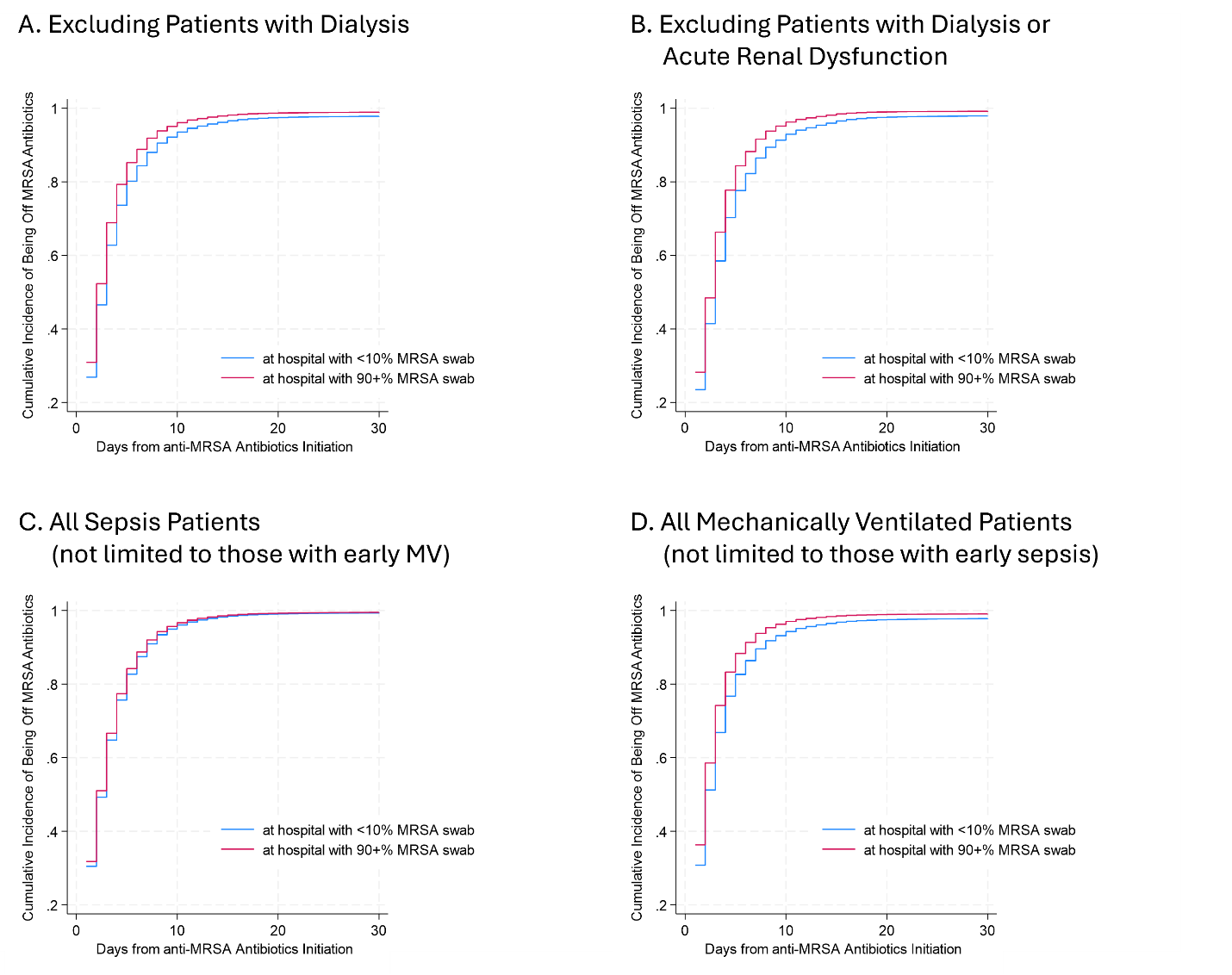


CI: confidence interval; MRSA: methicillin-resistant *Staphylococcus aureus*; MV: mechanical ventilation; SHR: sub-hazard ratio

*a* Using multivariable competing risks models; Panel A: SHR = 1.18 (95% CI: 1.05, 1.33), p=0.007; Panel B: SHR = 1.24 (1.08, 1.41), p=0.002; Panel C: SHR 1.05 (0.95, 1.16), p=0.31; Panel D: SHR = 1.23 (1.07, 1.41), p=0.003

eTable 8. Multivariable Time-to-Event Models Using Sensitivity Cohorts.*^a^*

|  | Excluding Dialysis Patients | | Excluding Dialysis & Acute Renal Dysfunction Patients | | Expanding to all Early Sepsis (+/- Early MV) | | Expanding to all Early MV (+/- Early Sepsis) | |
| --- | --- | --- | --- | --- | --- | --- | --- | --- |
|  | SHR (95% CI) | p-value | SHR (95% CI) | p-value | SHR (95% CI) | p-value | SHR (95% CI) | p-value |
| In hospital with ≥90% (vs <10%) early MRSA nasal swab use | 1.18 (1.05,1.33) | 0.007 | 1.24 (1.08,1.41) | 0.002 | 1.05 (0.95,1.16) | 0.31 | 1.23 (1.07,1.41) | 0.003 |
|  |  |  |  |  |  |  |  |  |
| Age (per 1 year) | 1.00 (1.00,1.01) | <0.001 | 1.00 (1.00,1.00) | <0.001 | 1.00 (1.00,1.01) | <0.001 | 1.00 (1.00,1.00) | <0.001 |
| Female gender | 1.10 (1.08,1.12) | <0.001 | 1.10 (1.07,1.14) | <0.001 | 1.10 (1.09,1.11) | <0.001 | 1.07 (1.05,1.08) | <0.001 |
| Race |  |  |  |  |  |  |  |  |
| White | ref |  | ref |  | ref |  | ref |  |
| Black | 0.99 (0.94,1.05) | 0.78 | 1.00 (0.92,1.08) | 0.95 | 1.04 (1.01,1.06) | 0.005 | 1.00 (0.96,1.04) | 0.89 |
| Other/unknown | 1.00 (0.95,1.05) | 1.00 | 1.01 (0.94,1.09) | 0.78 | 1.01 (0.98,1.05) | 0.46 | 0.98 (0.94,1.02) | 0.40 |
| Hispanic ethnicity | 0.99 (0.94,1.05) | 0.78 | 0.99 (0.92,1.06) | 0.69 | 1.03 (0.99,1.08) | 0.12 | 1.01 (0.96,1.07) | 0.67 |
| Payor |  |  |  |  |  |  |  |  |
| Private | ref |  | ref |  | ref |  | ref |  |
| Medicare | 1.00 (0.97,1.03) | 0.88 | 1.01 (0.96,1.05) | 0.77 | 1.00 (0.99,1.01) | 0.89 | 1.01 (0.98,1.04) | 0.61 |
| Medicaid | 0.96 (0.92,0.99) | 0.017 | 0.95 (0.91,1.00) | 0.07 | 0.98 (0.96,0.99) | 0.003 | 0.98 (0.95,1.01) | 0.19 |
| Other | 1.01 (0.97,1.06) | 0.54 | 1.01 (0.94,1.08) | 0.85 | 0.99 (0.97,1.01) | 0.53 | 1.03 (0.99,1.06) | 0.16 |
| Elixhauser comorbidities |  |  |  |  |  |  |  |  |
| Congestive heart failure | 1.01 (0.98,1.03) | 0.56 | 1.03 (0.99,1.08) | 0.13 | 1.01 (1.00,1.02) | 0.015 | 1.00 (0.99,1.02) | 0.64 |
| Valvular disease | 0.97 (0.94,1.01) | 0.15 | 0.99 (0.94,1.04) | 0.68 | 0.99 (0.98,0.99) | 0.002 | 1.01 (0.98,1.05) | 0.42 |
| Arrhythmia | 0.98 (0.96,1.00) | 0.07 | 1.00 (0.98,1.03) | 0.91 | 0.97 (0.96,0.97) | <0.001 | 0.98 (0.97,1.00) | 0.018 |
| Hypertension, uncomplicated | 0.97 (0.94,1.00) | 0.034 | 0.96 (0.92,0.99) | 0.023 | 1.00 (0.99,1.01) | 0.88 | 0.99 (0.96,1.02) | 0.41 |
| Hypertension, complicated | 1.01 (0.97,1.04) | 0.73 | 1.00 (0.95,1.05) | 0.87 | 1.02 (1.01,1.03) | 0.001 | 1.01 (0.98,1.04) | 0.39 |
| Peripheral vascular disease | 1.00 (0.97,1.03) | 0.96 | 0.99 (0.94,1.04) | 0.62 | 0.96 (0.95,0.98) | <0.001 | 1.02 (1.00,1.05) | 0.10 |
| Pulmonary disease | 1.02 (1.00,1.04) | 0.11 | 1.03 (0.99,1.06) | 0.10 | 1.00 (0.99,1.01) | 0.85 | 0.99 (0.97,1.00) | 0.11 |
| Pulmonary circulatory disorders | 0.94 (0.91,0.97) | <0.001 | 0.96 (0.91,1.01) | 0.13 | 0.96 (0.95,0.98) | <0.001 | 0.95 (0.93,0.98) | 0.001 |
| Diabetes mellitus, uncomplicated | 1.01 (0.98,1.04) | 0.57 | 1.02 (0.98,1.07) | 0.36 | 1.02 (1.00,1.03) | 0.009 | 1.01 (0.99,1.03) | 0.42 |
| Diabetes mellitus, complicated | 0.96 (0.95,0.98) | <0.001 | 0.96 (0.93,1.00) | 0.033 | 0.95 (0.94,0.95) | <0.001 | 0.97 (0.96,0.99) | <0.001 |
| Hypothyroidism | 0.97 (0.95,0.99) | 0.014 | 0.96 (0.93,1.00) | 0.030 | 1.00 (0.99,1.00) | 0.49 | 0.98 (0.96,0.99) | 0.010 |
| Rheumatoid arthritis / collagen vascular disease | 0.99 (0.95,1.03) | 0.59 | 0.97 (0.90,1.04) | 0.35 | 0.98 (0.96,0.99) | 0.001 | 0.97 (0.93,1.01) | 0.12 |
| AIDS/HIV | 1.05 (0.95,1.17) | 0.34 | 1.11 (0.95,1.29) | 0.20 | 1.08 (1.05,1.11) | <0.001 | 1.02 (0.94,1.11) | 0.62 |
| Lymphoma | 0.94 (0.86,1.02) | 0.15 | 1.02 (0.90,1.16) | 0.77 | 0.94 (0.93,0.96) | <0.001 | 0.91 (0.85,0.97) | 0.006 |
| Metastatic cancer | 0.94 (0.88,1.00) | 0.07 | 0.95 (0.87,1.03) | 0.22 | 0.97 (0.95,0.98) | <0.001 | 0.95 (0.91,1.00) | 0.06 |
| Tumor (no metastases) | 1.02 (0.97,1.07) | 0.51 | 0.99 (0.93,1.06) | 0.75 | 0.98 (0.97,1.00) | 0.034 | 1.00 (0.95,1.04) | 0.84 |
| Renal failure | 1.14 (1.10,1.18) | <0.001 | 1.18 (1.11,1.25) | <0.001 | 1.20 (1.19,1.22) | <0.001 | 1.19 (1.16,1.23) | <0.001 |
| Liver disease | 1.01 (0.98,1.04) | 0.58 | 1.03 (0.97,1.08) | 0.32 | 1.01 (1.00,1.02) | 0.046 | 1.00 (0.97,1.02) | 0.81 |
| Peptic ulcer disease | 0.96 (0.89,1.03) | 0.24 | 0.93 (0.83,1.04) | 0.21 | 1.02 (1.00,1.05) | 0.07 | 0.99 (0.94,1.04) | 0.72 |
| Paralysis | 0.98 (0.94,1.03) | 0.40 | 0.96 (0.91,1.02) | 0.18 | 0.95 (0.93,0.96) | <0.001 | 0.97 (0.94,1.01) | 0.12 |
| Other neurologic disorder | 0.99 (0.95,1.02) | 0.38 | 0.99 (0.95,1.04) | 0.70 | 1.00 (0.99,1.01) | 0.69 | 1.01 (0.98,1.03) | 0.61 |
| Blood loss anemia | 0.96 (0.89,1.04) | 0.35 | 0.99 (0.87,1.12) | 0.85 | 0.96 (0.94,0.99) | 0.002 | 0.96 (0.90,1.02) | 0.17 |
| Deficiency anemia | 0.98 (0.94,1.02) | 0.35 | 0.95 (0.90,1.00) | 0.06 | 0.97 (0.96,0.98) | <0.001 | 0.96 (0.93,0.99) | 0.022 |
| Coagulopathy | 0.97 (0.91,1.03) | 0.33 | 1.04 (0.91,1.18) | 0.59 | 0.99 (0.97,1.01) | 0.38 | 1.00 (0.95,1.05) | 1.00 |
| Obesity | 0.97 (0.94,1.00) | 0.024 | 0.94 (0.89,0.98) | 0.01 | 0.97 (0.96,0.99) | <0.001 | 0.97 (0.95,0.99) | 0.001 |
| Weight loss | 0.94 (0.91,0.96) | <0.001 | 0.92 (0.89,0.96) | <0.001 | 0.92 (0.91,0.94) | <0.001 | 0.93 (0.91,0.95) | <0.001 |
| Electrolyte disorder | 0.97 (0.94,1.00) | 0.025 | 0.97 (0.93,1.01) | 0.18 | 0.99 (0.98,1.00) | 0.011 | 0.95 (0.92,0.97) | <0.001 |
| Depression | 0.98 (0.96,1.01) | 0.20 | 1.00 (0.96,1.04) | 1.00 | 0.99 (0.97,1.00) | 0.047 | 0.98 (0.96,1.01) | 0.18 |
| Psychoses | 0.95 (0.90,1.00) | 0.05 | 0.98 (0.91,1.06) | 0.69 | 0.97 (0.95,0.99) | 0.004 | 0.94 (0.90,0.98) | 0.003 |
| Alcohol abuse | 1.07 (1.03,1.11) | 0.001 | 1.09 (1.03,1.16) | 0.003 | 1.02 (1.01,1.03) | 0.003 | 1.07 (1.04,1.10) | <0.001 |
| Drug abuse | 0.95 (0.91,1.00) | 0.048 | 0.95 (0.90,1.01) | 0.10 | 0.93 (0.91,0.96) | <0.001 | 0.97 (0.93,1.00) | 0.09 |
| Major surgery | 0.91 (0.88,0.94) | <0.001 | 0.89 (0.85,0.93) | <0.001 | 0.80 (0.79,0.81) | <0.001 | 1.01 (0.98,1.03) | 0.61 |
| Resource use |  |  |  |  |  |  |  |  |
| Vasopressors by HD 2 | 1.00 (0.97,1.04) | 0.86 | 0.94 (0.90,0.98) | 0.005 | 1.00 (0.98,1.02) | 0.94 | 1.02 (0.99,1.05) | 0.12 |
| Dialysis by HD 2 | omitted |  | omitted |  | 1.51 (1.46,1.56) | <0.001 | 1.31 (1.24,1.38) | <0.001 |
| Acute organ dysfunctions |  |  |  |  |  |  |  |  |
| Renal | 1.13 (1.10,1.16) | <0.001 | omitted |  | 1.04 (1.03,1.06) | <0.001 | 1.06 (1.04,1.08) | <0.001 |
| Neurologic | 1.01 (0.98,1.04) | 0.63 | 0.98 (0.94,1.03) | 0.44 | 1.00 (0.99,1.02) | 0.50 | 0.99 (0.97,1.02) | 0.52 |
| Liver | 1.06 (1.01,1.11) | 0.025 | 0.93 (0.83,1.04) | 0.21 | 1.03 (1.00,1.06) | 0.044 | 1.03 (1.00,1.07) | 0.09 |
| Hematologic | 1.04 (0.97,1.11) | 0.33 | 0.93 (0.81,1.07) | 0.33 | 1.01 (0.98,1.03) | 0.60 | 1.00 (0.94,1.06) | 0.98 |
| Cardiovascular | 0.93 (0.91,0.96) | <0.001 | 0.94 (0.91,0.98) | 0.002 | 1.00 (0.99,1.01) | 0.91 | 0.91 (0.89,0.93) | <0.001 |
| Discharge year |  |  |  |  |  |  |  |  |
| 2016 | ref |  | ref |  | ref |  | ref |  |
| 2017 | 1.01 (0.96,1.06) | 0.62 | 0.98 (0.92,1.04) | 0.43 | 1.04 (1.02,1.06) | <0.001 | 1.00 (0.95,1.05) | 0.90 |
| 2018 | 1.03 (0.97,1.10) | 0.33 | 1.02 (0.95,1.10) | 0.52 | 1.06 (1.03,1.09) | <0.001 | 1.01 (0.95,1.07) | 0.79 |
| 2019 | 1.06 (0.99,1.13) | 0.10 | 1.08 (1.00,1.16) | 0.04 | 1.10 (1.06,1.13) | <0.001 | 1.04 (0.98,1.10) | 0.24 |
| 2020 | 1.07 (1.00,1.14) | 0.05 | 1.04 (0.96,1.12) | 0.38 | 1.12 (1.08,1.15) | <0.001 | 1.05 (0.99,1.12) | 0.11 |
| 2021 | 1.08 (1.01,1.15) | 0.021 | 1.12 (1.03,1.22) | 0.011 | 1.12 (1.08,1.16) | <0.001 | 1.06 (1.00,1.13) | 0.05 |
| Teaching hospital | 1.03 (0.92,1.16) | 0.59 | 1.02 (0.91,1.14) | 0.69 | 1.02 (0.95,1.09) | 0.53 | 1.00 (0.89,1.13) | 0.95 |
| Hospital bed # |  |  |  |  |  |  |  |  |
| 500+ | ref |  | ref |  | ref |  | ref |  |
| 400-499 | 1.07 (0.91,1.27) | 0.41 | 1.08 (0.89,1.30) | 0.45 | 0.91 (0.83,1.01) | 0.07 | 0.99 (0.84,1.17) | 0.90 |
| 300-399 | 0.90 (0.80,1.01) | 0.08 | 0.89 (0.79,1.00) | 0.05 | 0.90 (0.82,0.98) | 0.014 | 0.89 (0.79,1.00) | 0.049 |
| 200-299 | 1.02 (0.88,1.18) | 0.80 | 0.98 (0.85,1.14) | 0.79 | 0.91 (0.83,1.00) | 0.06 | 1.00 (0.85,1.16) | 0.95 |
| 100-199 | 1.01 (0.87,1.17) | 0.92 | 1.02 (0.88,1.19) | 0.78 | 0.93 (0.86,1.01) | 0.11 | 0.97 (0.82,1.13) | 0.67 |
| 0-99 | 1.09 (0.85,1.41) | 0.51 | 1.16 (0.86,1.58) | 0.33 | 0.91 (0.81,1.03) | 0.14 | 1.09 (0.88,1.36) | 0.42 |
| Hospital region |  |  |  |  |  |  |  |  |
| Midwest | ref |  | ref |  | ref |  | ref |  |
| Northeast | 1.39 (1.14,1.69) | 0.001 | 1.42 (1.15,1.76) | 0.001 | 1.08 (0.97,1.21) | 0.16 | 1.30 (1.11,1.51) | 0.001 |
| South | 1.16 (1.06,1.27) | 0.002 | 1.19 (1.07,1.31) | 0.001 | 1.03 (0.95,1.11) | 0.53 | 1.13 (1.02,1.26) | 0.019 |
| West | 1.03 (0.87,1.21) | 0.76 | 1.02 (0.85,1.22) | 0.85 | 1.01 (0.91,1.11) | 0.86 | 0.96 (0.82,1.12) | 0.59 |
| Urban hospital | 1.15 (0.96,1.39) | 0.14 | 1.16 (0.96,1.42) | 0.13 | 1.11 (1.01,1.22) | 0.037 | 1.25 (1.07,1.46) | 0.006 |

HD: hospital day; MRSA: methicillin-resistant *Staphylococcus aureus*; ref: reference; SHR: sub-hazard ratio

*a* Using multivariable competing risks models

eFigure 5. Association of Hospital-Level Early MRSA Nasal Swab Use and Discontinuation of Piperacillin-Tazobactam and/or Cefepime.*^a^*


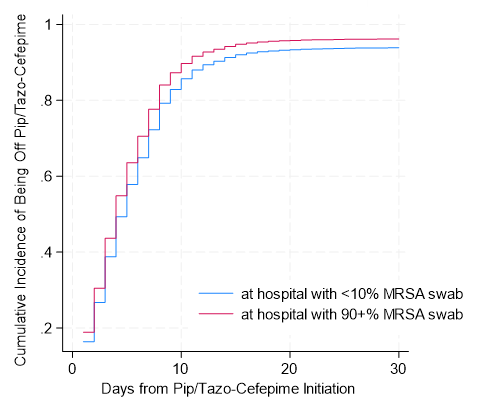


CI: confidence interval; MRSA: methicillin-resistant *Staphylococcus aureus*; Pip/Tazo: Piperacillin-Tazobactam; SHR: sub-hazard ratio

*a* Using multivariable competing risks model; SHR = 1.16 (95% CI: 1.01, 1.34), p=0.035

eTable 9. Multivariable Time-to-Event Model for “Negative Outcome Control” of Piperacillin-Tazobactam and/or Cefepime.*^a^*

|  | Discontinuation of Piperacillin-Tazobactam and/or Cefepime | |
| --- | --- | --- |
|  | SHR (95% CI) | p-value |
| In hospital with ≥90% (vs <10%) early MRSA nasal swab use | 1.16 (1.01,1.34) | 0.035 |
|  |  |  |
| Age (per 1 year) | 1.00 (1.00,1.00) | 0.36 |
| Female gender | 1.04 (1.02,1.06) | <0.001 |
| Race |  |  |
| White | ref |  |
| Black | 0.96 (0.92,1.01) | 0.08 |
| Other/unknown | 0.96 (0.91,1.02) | 0.24 |
| Hispanic ethnicity | 0.95 (0.91,1.00) | 0.032 |
| Payor |  |  |
| Private | ref |  |
| Medicare | 0.99 (0.96,1.02) | 0.64 |
| Medicaid | 1.00 (0.96,1.03) | 0.80 |
| Other | 1.05 (1.01,1.10) | 0.021 |
| Elix. comorbidities |  |  |
| Congestive heart failure | 1.02 (0.99,1.05) | 0.30 |
| Valvular disease | 1.01 (0.98,1.05) | 0.50 |
| Arrhythmia | 0.95 (0.94,0.97) | <0.001 |
| Hypertension, uncomplicated | 0.99 (0.96,1.03) | 0.73 |
| Hypertension, complicated | 1.01 (0.98,1.05) | 0.42 |
| Peripheral vascular disease | 0.98 (0.95,1.01) | 0.29 |
| Pulmonary disease | 0.99 (0.97,1.01) | 0.31 |
| Pulmonary circulatory disorders | 0.99 (0.96,1.02) | 0.38 |
| Diabetes mellitus, uncomplicated | 1.02 (0.99,1.04) | 0.27 |
| Diabetes mellitus, complicated | 1.01 (0.99,1.03) | 0.27 |
| Hypothyroidism | 1.02 (1.00,1.04) | 0.11 |
| Rheumatoid arthritis / collagen vascular disease | 1.00 (0.95,1.05) | 0.96 |
| AIDS/HIV | 1.03 (0.92,1.15) | 0.59 |
| Lymphoma | 1.04 (0.95,1.14) | 0.35 |
| Metastatic cancer | 0.93 (0.88,0.98) | 0.009 |
| Tumor (no metastases) | 0.91 (0.87,0.95) | <0.001 |
| Renal failure | 0.99 (0.96,1.02) | 0.46 |
| Liver disease | 0.98 (0.95,1.02) | 0.31 |
| Peptic ulcer disease | 0.99 (0.90,1.07) | 0.75 |
| Paralysis | 1.04 (0.99,1.09) | 0.09 |
| Other neurologic disorder | 0.98 (0.95,1.01) | 0.21 |
| Blood loss anemia | 0.90 (0.83,0.97) | 0.004 |
| Deficiency anemia | 1.00 (0.97,1.04) | 0.94 |
| Coagulopathy | 0.99 (0.92,1.07) | 0.81 |
| Obesity | 0.98 (0.95,1.00) | 0.08 |
| Weight loss | 0.98 (0.96,1.01) | 0.28 |
| Electrolyte disorder | 0.92 (0.90,0.95) | <0.001 |
| Depression | 1.01 (0.98,1.04) | 0.64 |
| Psychoses | 1.00 (0.95,1.05) | 0.90 |
| Alcohol abuse | 1.04 (1.01,1.07) | 0.014 |
| Drug abuse | 1.12 (1.08,1.16) | <0.001 |
| Major surgery | 0.91 (0.89,0.94) | <0.001 |
| Resource use |  |  |
| Vasopressors by HD 2 | 0.92 (0.89,0.95) | <0.001 |
| Dialysis by HD 2 | 1.07 (1.02,1.13) | 0.007 |
| Acute organ dysfunc |  |  |
| Renal | 0.96 (0.94,0.99) | 0.003 |
| Neurologic | 0.97 (0.94,1.01) | 0.12 |
| Liver | 0.90 (0.87,0.94) | <0.001 |
| Hematologic | 1.00 (0.92,1.07) | 0.92 |
| Cardiovascular | 0.91 (0.89,0.94) | <0.001 |
| Discharge year |  |  |
| 2016 | ref |  |
| 2017 | 0.98 (0.94,1.02) | 0.38 |
| 2018 | 1.00 (0.95,1.05) | 1.00 |
| 2019 | 1.01 (0.96,1.06) | 0.80 |
| 2020 | 0.99 (0.93,1.06) | 0.81 |
| 2021 | 1.04 (0.97,1.10) | 0.26 |
| Teaching hospital | 1.02 (0.90,1.16) | 0.73 |
| Hospital bed # |  |  |
| 500+ | ref |  |
| 400-499 | 1.02 (0.86,1.21) | 0.80 |
| 300-399 | 0.94 (0.83,1.06) | 0.33 |
| 200-299 | 1.00 (0.86,1.16) | 0.96 |
| 100-199 | 0.98 (0.85,1.15) | 0.84 |
| 0-99 | 1.05 (0.84,1.33) | 0.67 |
| Hospital region |  |  |
| Midwest | ref |  |
| Northeast | 1.26 (1.00,1.59) | 0.05 |
| South | 1.10 (0.97,1.25) | 0.14 |
| West | 0.97 (0.80,1.17) | 0.72 |
| Urban hospital | 1.08 (0.90,1.28) | 0.41 |

Elix.: Elixhauser; dysfunc.: dysfunction; HD: hospital day; HR: hazard ratio; MRSA: methicillin-resistant *Staphylococcus aureus*; ref: reference; SHR: sub-hazard ratio

*a* Using a multivariable competing risks model. The cohort included all patients cared for in hospitals in the primary outcomes cohort since 2016 who received piperacillin-tazobactam and/or cefepime by hospital day 2: 32,471 in 67 hospitals with <10% early MRSA nasal swab use and 9,509 in 33 hospitals with ≥90% early MRSA nasal swab use. Calculations of % early MRSA nasal swab use were based on the primary cohort of patients receiving anti-MRSA antibiotics by hospital day 2.
